# Mitsugumin 53 drives stem cell differentiation easing intestinal injury and inflammation

**DOI:** 10.1038/s41392-025-02268-x

**Published:** 2025-06-11

**Authors:** Yumeng Pei, Meng Fang, Hong-Kun Wu, Qionghua Cui, Li Quan, Xiaochuan Li, Keyi Zhang, Peng Xie, Peng Jiang, Yuan Liu, Meimei Huang, Fengxiang Lv, Xiaomin Hu, Ye-Guang Chen, Xinli Hu, Rui-Ping Xiao

**Affiliations:** 1 State Key Laboratory of Membrane Biology, Institute of Molecular Medicine, College of Future Technology, Peking University, 100871 Beijing, China; 2 Peking-Tsinghua Center for Life Sciences, 100871 Beijing, China; 3 Department of Hepatobiliary and Pancreatic Surgery, The First Affiliated Hospital, Zhejiang University School of Medicine, Hangzhou, 310006 China; 4 Zhejiang Provincial Key Laboratory of Pancreatic Disease, Hangzhou, 310006 China; 5 The State Key Laboratory of Membrane Biology, Tsinghua-Peking Center for Life Sciences, School of Life Sciences, Tsinghua University, Beijing, 100084 China; 6 Beijing City Key Laboratory of Cardiometabolic Molecular Medicine, Peking University, 100871 Beijing, China; 7 State Key Laboratory for Complex, Severe, and Rare Diseases, Peking Union Medical College Hospital, Chinese Academy of Medical Sciences & Peking Union Medical College, 100730 Beijing, China

**Keywords:** Gastrointestinal diseases, Intestinal stem cells

## Abstract

Emerging evidence suggests that priming intestinal stem cells (ISCs) towards secretory progenitor cells is beneficial for maintaining gut homeostasis against inflammatory bowel disease (IBD). However, the mechanism driving such biased lineage commitment remains elusive. Here we show that MG53, also named as TRIM72, prompts ISCs to secretory lineages via upregulating peroxisome proliferator-activated receptor α (PPARα), thus maintaining intestinal epithelium integrity against noxious insults. Using genetic mouse models, we found that MG53 deficiency leads to exacerbated intestinal damage caused by various injuries in mice, whereas MG53 overexpression in ISCs is sufficient to ameliorate such damage. Mechanistically, MG53 promoted asymmetric division of ISCs to generate more progenitor cells of secretory lineages via activating PPARα signaling. Specifically, MG53 overexpression induced PPARα expression at transcriptional level and concomitantly increased PPARα activity by elevating the contents of a panel of unsaturated fatty acids in the intestine that serve as potent endogenous agonists of PPARα. Furthermore, genetic ablation or pharmacological inhibition of PPARα abolished the protective effects of MG53. These findings reveal a crucial role of MG53-PPARα axis in driving the secretory lineage commitment of ISCs, especially during injury response, highlighting the important therapeutic potential of targeting MG53-PPARα signaling for IBD treatment and marking PPARα agonists as novel therapies for IBD caused by various etiologies.

## Introduction

Inflammatory bowel disease (IBD), including Crohn’s disease and ulcerative colitis, poses a significant threat to human health. The major clinical manifestation of IBD is chronic inflammation of the digestive tract. The recurrent inflammation leads to a range of symptoms such as diarrhea, abdominal pain, and weight loss. Therefore, the current therapeutic regiments primarily targets various inflammatory cascades, such as drugs targeting TNF-α (infliximab and adalimumab), integrin (natalizumab and vedolizumab), Janus kinase (tofacitinib), and sphingosine-1-phosphate (S1P)-receptor (ozanimod).^1,2^ Regardless of strenuous financial burden, these drugs increase the susceptibility to pathogen infections in addition to various other life-threatening side effects.^1–4^ More importantly, the ultimate goal of IBD treatment is to restore the structural and functional integrity of intestinal epithelium, while management of recurrent inflammatory condition is only one part of the solution. Thus, existing treatment targeting inflammation can only alleviate the symptoms, but cannot fully address the unmet medical needs in IBD.

In fact, even under physiological conditions, the proper functioning of the intestinal track relies on the continuous replacement of intestinal epithelial cells by fast proliferating progenitors.^5^ These progenitor cells are derived from intestinal stem cells (ISCs). The commitment to either the absorptive or secretory progenitor cell is the initial step in ISC differentiation and dictates whether the ISCs differentiate into mature absorptive cells (enterocytes) or secretory cells (e.g., tuft cells, goblet cells, enteroendocrine cells, and Paneth cells).^6^ The ISC fate decision is influenced by various signals in the ISC niche, including metabolic cues, which strongly favors the absorptive lineage.^5,7,8^ Nevertheless, studies show that the secretory lineage cells can revert to the state of stem cell under stress or pathological conditions.^9^ In response to injury, these cells can exit dormant status and undergo rapid proliferation. They can then differentiate into multiple lineages encompassing the major epithelial cell types.^10^ The plasticity of secretory progenitor cells relies on phosphorylation of ATOH1, a key regulator of secretory lineage commitment. Thus, prevention of ATOH1 phosphorylation impairs intestinal regeneration in response to injury.^11^ Moreover, the induction of ATOH1 expression and the resultant differentiation towards secretory lineages, is critical for the integrity of the mucosa following damage.^12^ Upon irradiation, even the mature Paneth cells can reinitiate cell cycle and acquire stem-cell characteristics, contributing to epithelial replenishment following stem cell loss.^13^ These studies suggest that tipping the balance towards secretory lineages can reduce intestinal damage and accelerate epithelial regeneration of intestines to better cope with various challenges, raising the possibility of modulating ISC fate to ameliorate inflammation-induced intestinal injury in IBD.

Mitsugumin 53 (MG53) is a multifunctional protein belonging to tripartite motif-containing protein family (also known as TRIM72). It functions as an E3 ligase to induce ubiquitination of insulin receptor and insulin receptor substrate 1, thereby facilitating their proteasomal degradation.^14,15^ It also acts as a transcriptional regulator of peroxisome proliferator-activated receptor α (PPARα) in the heart to enhance lipid utilization by cardiac myocytes.^16^ On the hand, MG53 plays a central role in cell membrane repair, serving as an essential component of repair machinery and protects multiple organs against acute injury.^17–19^ In particular, we have shown that MG53 protects mice against colorectal damage induced by azoxymethane (AOM)/dextran sodium sulfate (DSS) and resultant tumor growth.^20^ In this study, we seek to determine whether MG53 is essentially involved in intestinal repair in response to harmful stimuli and, if so, to reveal the underlying mechanism.

We observed that MG53 transgenic mice (MG53-TG) had better preserved intestinal structure following different damaging insults, while depletion of MG53 exacerbated the injuries. The advantage of MG53-TG was associated with an elongated transient amplifying (TA) zone in the intestine. Unexpectedly, single-cell RNA sequencing (scRNA-seq) revealed a decreased proportion of Lgr5^+^ stem cells, accompanied by an increase in cells belonging to secretory lineages. Further analysis suggested that PPARα signaling might be involved in the asymmetric division of ISCs induced by MG53 overexpression. Previous studies have shown that PPARα activation can alleviate IBD symptoms.^21,22^ A recent study reports that PPARα agonist protects mice against colitis by rescuing loss in ISCs, while the protective effects were negated in the intestinal-specific PPARα deficient mice.^23^ By manipulating PPARα signaling with genetic or pharmacological approaches, we have demonstrated that the protective effects of MG53 are mediated by PPARα. MG53 upregulates the transcription of PPARα and elevates the content of endogenous PPARα agonists, which in turn, drive the proliferation and secretory lineage commitment of ISCs. The expanded secretory progenitor cell population in MG53-TG mice helps maintain intestinal integrity by enabling rapid replacement of damaged cells following injury. These findings reveal the role of MG53- PPARα axis in intestinal protection and highlight the potential of PPARα agonists in treating intestinal injury, including IBD.

## Results

### MG53 overexpression ameliorates, while its deficiency worsens, intestinal injury in multiple mouse models of IBD

**Fig. 1:**
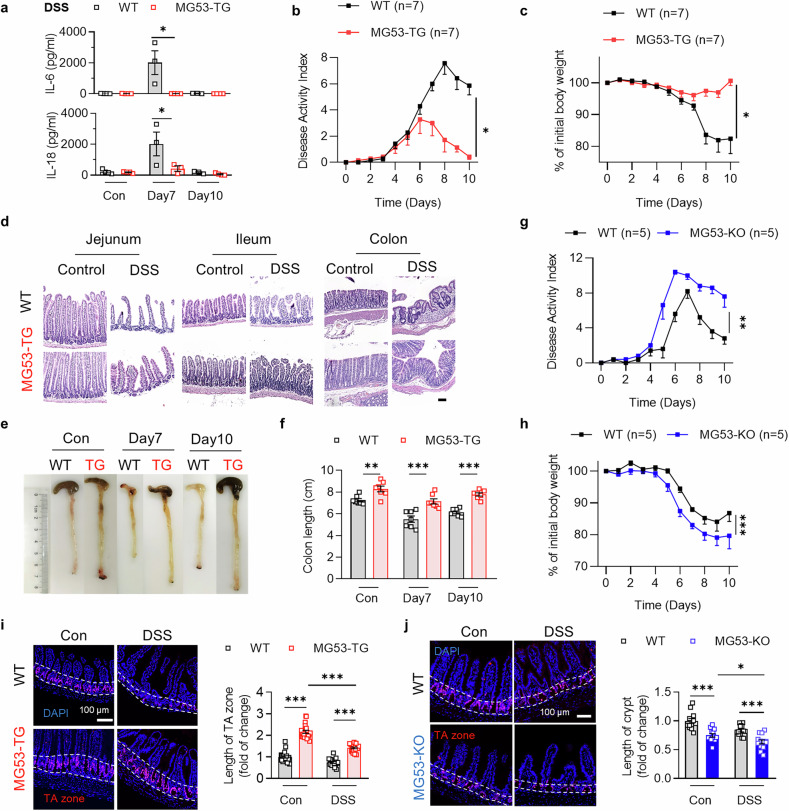
MG53 attenuates intestinal injury in mouse models. **a** Serum IL-6 and IL-18 levels of MG53-TG and their WT littermates at the indicated time points following DSS treatment. *n* = 3 for each group. Overexpression of MG53 attenuated DSS-induced IBD symptoms, including disease activity index (DAI; **b**), body weight change (**c**), disruption of intestinal structure as demonstrated by H&E staining of the jejunum, ileum, and colon (**d**; scale bar, 100 μm), and shortening of the colon as evidenced by the representative images (**e**) and statistic results of colon length (**f**) in MG53-TG and WT mice. *n* = 7 for each group. **g**, **h** Depletion of MG53 exacerbated DSS-induced IBD symptoms. The DAI (**g**) and body weight change (**h**) of MG53-KO and the WT littermates at the indicated time points after DSS challenge. *n* = 5 for each group. Immunofluorescence staining of Ki-67 (red) to indicate the TA zone and the statistic results of its length in MG53-TG (**i**, *n* = 20 for each group) or MG53-KO (**j**, *n* = 13 for each group) as compared with their corresponding WT littermates. The nuclei were indicated by DAPI staining (blue); scale bar, 100 μm. Normal distribution was confirmed by Shapiro–Wilk test. Data were analyzed using two tailed paired *t* test (**a**–**c**, **f**–**j**). All data were presented as mean ± s.e.m. *p < 0.05, **p < 0.01, and ***p < 0.001 as compared with the corresponding controls

To determine the function of MG53 in the intestinal epithelial repair, MG53-TG with global MG53 overexpression (Supplementary Fig. 1a) and their wild type littermates (WT) were administered 2.5% DSS to induce injury in the intestine (Supplementary Fig. 1b). DSS treatment did not alter MG53 mRNA levels in different intestinal segments of WT mice 7 days post DSS treatment (Supplementary Fig. 1c), while overexpression of MG53 in MG53-TG significantly attenuated DSS-induced increases in circulating IL-6 and IL-18 levels (Fig. 1a) as well as intestinal pro-inflammatory cytokine production (Supplementary Fig. 1d). Moreover, over the course of 7-day DSS treatment, overexpression of MG53 lowered the disease activity index (DAI) which was evaluated by stool consistency and presence of rectal bleeding, as well as loss in body weight (Fig. 1b). The body weight was better maintained in MG53-TG mice (Fig. 1c). After switching back to plain drinking water for 3 days, MG53-TG recovered faster with better-preserved intestinal architecture and less colon shortening compared with their WT littermates (Fig. 1d–f).

Similar protective effects were observed in the LPS-induced intestinal inflammation.^24^ The MG53-TG mice displayed improved intestinal structure following LPS treatment (Supplementary Fig. 1e). Under both DSS and LPS treatments, MG53 overexpression markedly mitigated apoptotic cell death (Supplementary Fig. 1f, g). Consistently, propidium iodide (PI) staining of crypt organoids showed that MG53 diminished DOX-induced cell death in the organoids derived from MG53-TG mice (Supplementary Fig. 1h).

In contrast, MG53 deficient mice (MG53-KO) exhibited exaggerated body weight loss and disease severity index, along with more severe colon shortening and worsened injury of intestinal barrier in response to DSS challenge (Fig. 1g, h and Supplementary Fig. 2). Collectively, these data indicate that MG53 protects intestinal integrity against intestinal injury and ensuing inflammation.

### MG53 overexpression results in an elongated TA zone

To understand how MG53 exerts the protective effect, we first tested whether gut-microbiota contribute to the improvement of intestinal condition in MG53-TG mice. To this end, gut microbiota were depleted by treating the mice with a broad-spectrum antibiotics ABX. Following the treatment, the curative benefit of MG53 still existed (Supplementary Fig. 3a, b), indicating that MG53 improves survival outcome in response to LPS challenge independent of microbiota.

The efficient recovery of MG53-TG mice from DSS-instigated intestinal damage prompted us to investigate the intestinal epithelial regeneration after damage. Intestinal self-renewal primarily depends on the dormant stem cells at the crypt base columnar zone and progenitor cells in the TA zone. TA zone is enriched with Ki-67^+^ fast proliferating and amplifying progenitor cells. The immunofluorescence staining of Ki-67 of the terminal ileum showed that MG53-TG mice had an expanded TA zone both at basal condition and after challenged with DSS or LPS (Fig. 1i and Supplementary Fig. 3c). In contrast, MG53-KO mice displayed a shorter TA zone than their WT littermates (Fig. 1j).

When the mice were subjected to γ irradiation to deplete the proliferating cells, the difference in the length of TA zone between WT and MG53-TG was erased (Supplementary Fig. 4a, b).^25,26^ There was no difference between MG53-TG and their WT littermates in terms of the amount of apoptotic cells, the degree of intestinal injury, or the survival outcome following irradiation (Supplementary Fig. 4c–e). γ irradiation also eliminated the difference between the MG53-KO and their WT control (Supplementary Fig. 4f, g). Importantly, DSS accelerated irradiation-induced death, but MG53-TG exhibited no survival advantage over WT (Supplementary Fig. 4e). Moreover, γ irradiation abolished alleviation of DSS-induced body weight loss and reduction in DAI in the MG53-TG (Supplementary Fig. 4h, i). These findings demonstrate that the progenitor cells in the elongated TA zone are essential for MG53-mediated amelioration of intestinal damage.

### MG53 promotes ISC secretory lineage commitment

**Fig. 2:**
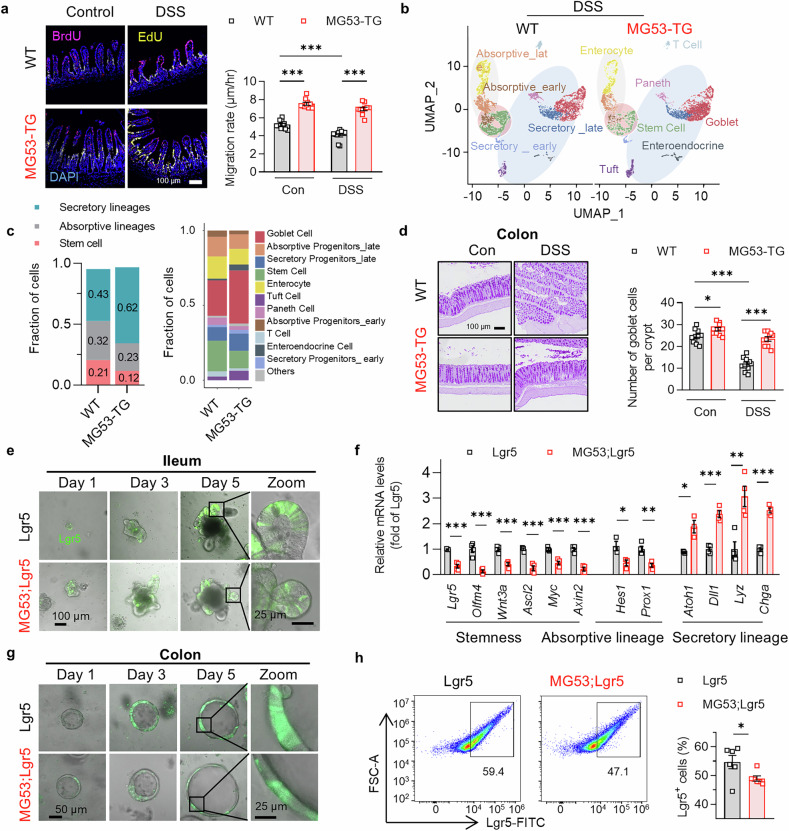
MG53 overexpression promotes secretory lineage commitment. **a** Immunofluorescence staining of BrdU (red) and EdU (yellow) in the ileum of MG53-TG and their WT littermates subjected to DSS treatment and administered BrdU 48 h and EdU 24 h before euthanasia. *n* = 10 for each group. Scale bar, 100 μm. **b** UMAP of scRNA-seq of Epcam^+^CD45^−^ intestinal epithelial cells collected from MG53-TG and their WT littermates on Day 7 of DSS treatment, *n* = 5 for each group. **c** Proportions of stem, secretory, and absorptive cells in general and each particular cell clusters. **d** Representative images and statistic results of PAS staining of goblet cells of MG53-TG and WT mice in the colon. *n* = 10 for each group. Scale bar, 100 μm. **e** Representative images of intestinal organoids derived from MG53;Lgr5 mice and their Lgr5 littermates at the indicated time points. **f** Relative mRNA levels of the marker genes in the transcriptome analysis of the crypt organoids. *n* = 4 for each group. **g** Representative images of colonic organoids derived from MG53;Lgr5 mice and their Lgr5 littermates at the indicated time points. **h** Representative FACS profiles and statistic results of Lgr5^+^ cells in the colonic organoids derived from MG53;Lgr5 and the control Lgr5 mice. *n* = 6 for each group. Normal distribution was confirmed by Shapiro–Wilk test. Data were analyzed using two tailed paired *t* test (**a**, **d**, **f**, and **h**), and were presented as mean ± s.e.m. *p < 0.05, **p < 0.01, and ***p < 0.001 as compared with the corresponding controls

To precisely assess the effect of MG53 in the expansion of TA zone, we performed dual-labeled tracing of intestinal proliferating cells with 5-bromo-2′-deoxyuridine (BrdU) and 5-ethynyl-2′-deoxyuridine (EdU)^27^ to monitor the efficiency of stem and progenitor cell proliferation, differentiation, and maturation, as evidenced by their migration speed along the crypt-villus axis. Dividing the distance between the fronts of BrdU-positive cells and EdU-positive cells by the time difference between the BrdU and EdU administration, we found that cell migration was indeed profoundly accelerated in the MG53-TG (Fig. 2a), implying that overexpression of MG53 promotes progenitor cell amplification which may account for the elongation of TA zone.

Next, we sought to delineate the mechanism underlying MG53-induced expansion of TA zone. Using scRNA-seq, we analyzed Epcam^+^CD45^−^ cells collected from the intestinal epithelium of the DSS-treated mice and identified 11 cell clusters with their specific marker genes and a few unidentified cells were grouped as “other” (Fig. 2b and Supplementary Fig. 5a). Surprisingly, despite the accelerated epithelial cell turnover, the proportion of stem cells was lower in the MG53-TG relative to control mice (Fig. 2b, c). In addition, the cells belong to the absorptive lineages were decreased, whereas the secretory lineage cells were increased (Fig. 2c). This was substantiated by comparing the scores of “stemness”, “absorptive”, and “secretory” properties of all the Epcam^+^CD45^−^ cells with or without MG53 overexpression (Supplementary Fig. 5b). Consistently, histological staining demonstrated that MG53-TG mice had more secretory PAS^+^ goblet cells and Lyz^+^ Paneth cells, particularly following DSS treatment (Fig. 2d and Supplementary Fig. 5c, d). In contrast, fewer goblet cells could be detected in the MG53-KO colons (Supplementary Fig. 5e).

Lgr5 marks stem cells in the intestine. To understand the effects of MG53, we overexpressed MG53 in the Lgr5-EGFP-IRES-CreERT2 knock-in mice (Lgr5)^6^ by crossing this line with MG53-TG (MG53;Lgr5), then examined the crypt organoids derived from MG53;Lgr5 or the control Lgr5 mice. MG53;Lgr5 intestinal organoids generated buds as early as day 1 when the Lgr5 organoids were still bud-free, and displayed more buds and larger size with prolonged culture (Fig. 2e and Supplementary Fig. 6a, b). In the organoids derived from MG53;Lgr5 mice, fluorescence-activated cell sorting (FACS) detected fewer Lgr5^+^ stem cells (Supplementary Fig. 6c). The transcriptome of the MG53;Lgr5 crypt organoids displayed an declined expression of marker genes of stemness and absorptive lineages, while elevated expression of those belonging to the secretory lineages (Fig. 2f). The reduced population of Lgr5^+^ cells was also observed in the colonic organoids from MG53;Lgr5 mice (Fig. 2g, h). In particular, the mRNA levels of absorptive lineage marker *Hes1* was reduced, whereas that of the secretory lineage marker *Atoh1* was significantly upregulated in the MG53-TG organoids (Supplementary Fig. 6d). While the Ki-67^+^ cells were increased (Supplementary Fig. 6e). Besides, in human intestinal epithelial cell HIEC-6, the expression of critical transcription factors regulating secretory lineage differentiation, such as *MUC2*, *SOX9*, and *SPDEF* were markedly enhanced by adenovirus-mediated MG53 overexpression under basal condition and after DSS exposure, whereas *PARP*, marker of apoptosis, was downregulated (Supplementary Fig. 6f). Collectively, these results demonstrate that MG53 promotes ISC differentiation towards secretory progenitor cells.

### PPARα signaling is enhanced by MG53 at transcriptional level

**Fig. 3:**
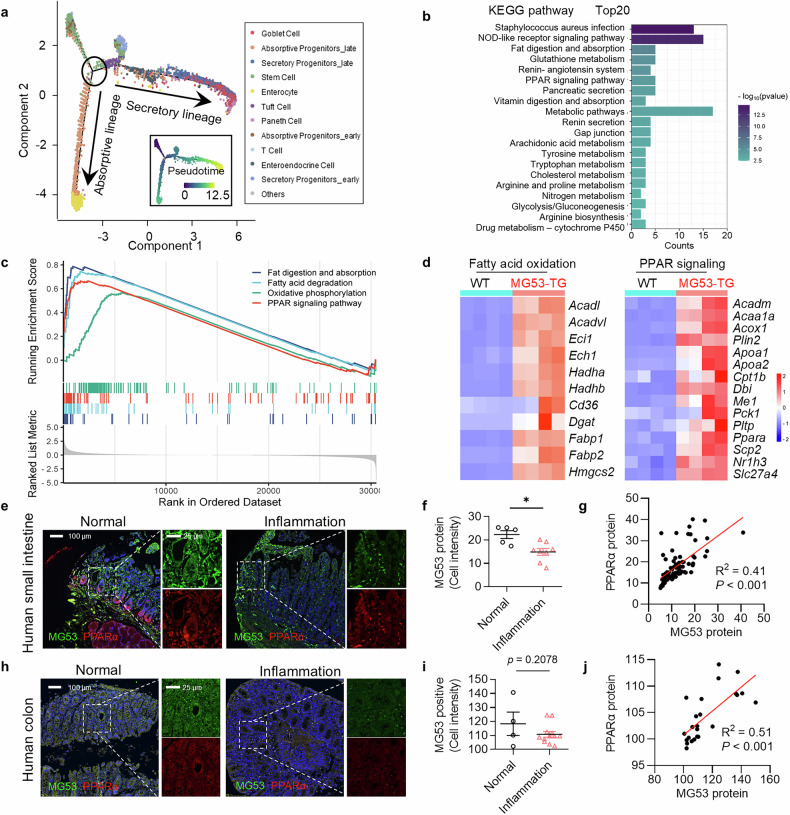
PPAR signaling is enhanced by MG53 overexpression. **a** Trajectory analysis of Epcam^+^CD45^−^ intestinal epithelial cells Using Monocle 2. **b** KEGG pathway enrichment analysis of the differentially expressed genes between the secretory and absorptive branches. GSEA results (**c**) and the heatmap of differentially expressed genes (**d**) related to fatty acid oxidation and PPAR signaling derived from the bulk RNAseq data of the intestinal organoids from MG53-TG and WT mice. *n* = 4 for each group. Representative images (**e**) and statistic results of signal intensity of the immunofluorescence staining of MG53 (green) and PPARα (red) determined by Aperio Scanscope scanner and Halo software (**f**), as well as the correlation of the levels of these two proteins (**g**) in the human small intestine of normal subjects (*n* = 5) and patients with intestinal inflammation (*n* = 8). Nuclei were stained with DAPI (blue); scale bars as indicated. Representative images (**h**) and statistic results of signal intensity of the immunofluorescence staining of MG53 (green) and PPARα (red) determined by Aperio Scanscope scanner and Halo software (**i**), as well as the correlation of the levels of these two proteins (**j**) in the human colon of normal subjects (*n* = 4) and patients with intestinal inflammation (*n* = 12). Normal distribution was confirmed by Shapiro–Wilk test. Data were analyzed using Mann–Whitney *U* test (**f**, **i**) and Pearson correlation analysis (**g**, **j**). Data were presented as mean ± s.e.m. *p < 0.05 as compared with the corresponding controls

Next, we analyzed the trajectory of ISC differentiation and searched for the pathway(s) that may influence the absorptive/secretory lineage decision-making using KEGG analysis (Fig. 3a, b). Besides inflammation-related pathways, several metabolic pathways were upregulated in the ISCs from DSS-treated MG53;Lgr5 compared with Lgr5 controls (Fig. 3b). The transcriptome of the crypt organoids was further examined by Gene Set Enrichment Analysis (GSEA), which showed that the genes differentially expressed were related to pathways of PPAR signaling and fatty acid metabolism (Fig. 3c, d), in line with the results of KEGG analysis of the scRNA-seq data.

Since our previous study has shown that MG53 acts as an important transcriptional regulator of PPARα in the heart,^16^ we hypothesized that PPARα may be involved in the MG53-mediated amelioration of intestinal damage. Indeed, the mRNA level of PPARα, but not PPARδ or PPARγ, was increased in the intestinal tract of MG53-TG compared to WT controls (Supplementary Fig. 7a). Accordingly, knocking down endogenous MG53 in HCT116 cells led to a significant decline of PPARα at transcriptional level without alterations in PPARδ or PPARγ expression (Supplementary Fig. 7b). Importantly, MG53 protein level was decreased in the human small intestine tissues with chronic inflammation, and trended lower in the inflammatory colon tissues (Fig. 3e, f, h, i), and the abundance of MG53 and PPARα was positively correlated in the human small intestines and colons (Fig. 3g, j and Supplementary Fig. 7c). To directly access the transcriptional regulation of PPARα by MG53 in intestine, we evaluated the *PPARA* promoter activity using luciferase reporter assay in HIEC-6 cells, and found a robust activation of *PPARA* promoter by MG53 overexpression (Supplementary Fig. 7d). These results strongly suggest that MG53 is a transcriptional regulator of PPARα in the intestine.

### MG53-PPARα axis promotes secretory lineage commitment via triggering asymmetric division of ISCs

**Fig. 4:**
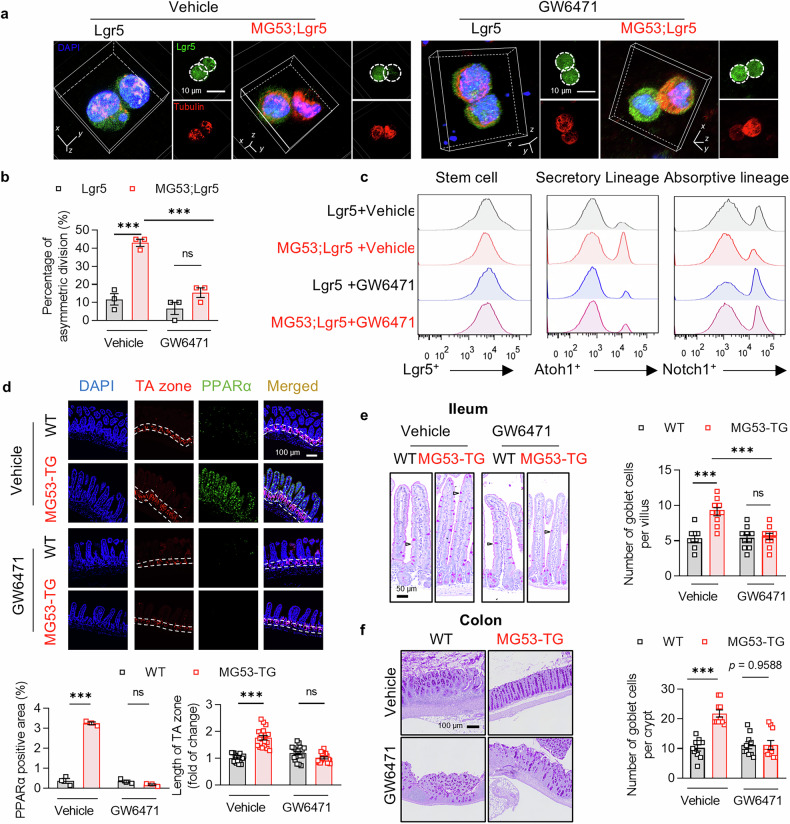
PPARα mediates the effect of MG53 in promoting asymmetric division of Lgr5^+^ cells. Representative images (**a**) and statistic results (**b**, *n* = 3 for each group) of asymmetric division of Lgr5^+^ cells with or without GW6471 (10 μM, 24 h) treatment. Lgr5^+^ cells were in green; all the cells were visualized by tubulin (red). Scale bar, 10 μm. **c** FACS analysis of Lgr5^+^, Atoh1^+^, and Notch1^+^ cells in the Lgr5 and MG53;Lgr5 organoids treated with GW6471 or vehicle. **d** Immunofluorescence staining of Ki-67 (red) and PPARα (green) and statistic results of PPARα positive cells (*n* = 3 for each group) and the length of TA zone (*n* = 18 for each group) in mice treated GW6471 or vehicle. Nuclei were stained with DAPI (blue); scale bar, 100 μm. Representative images and statistic results of PAS staining of goblet cells in the ileum (**e**; *n* = 10 for each group; TG, MG53-TG; scale bar, 50 μm) and colon (**f**; *n* = 10 for each group; scale bar, 100 μm). Normal distribution was confirmed by Shapiro–Wilk test. Data were analyzed using one-way ANOVA with Tukey post hoc test (**b**, **d**, **e**, and **f**) and were presented as mean ± s.e.m. ns not significant and ***p < 0.001 as compared with the corresponding controls

To understand the role of PPARα in the MG53-mediated biased differentiation towards secretory lineages, we monitored the division of Lgr5^+^ cells obtained from the crypts of MG53;Lgr5 or Lgr5 mice. Interestingly, with MG53 overexpressed, nearly half of the Lgr5^+^ cells underwent asymmetric division as one of their daughter cells lost GFP expression that was under the control of *Lgr5* promoter, while the asymmetrically divided cells were only about 10% in the control mice (Fig. 4a, b). This result indicated that MG53 overexpression suppresses the self-renewal of intestinal stem cells, which may account for the decreased Lgr5^+^ population in MG53-TG.

The scRNA-seq data implied that MG53 influences ISC differentiation via PPAR signaling. Indeed, it has been reported that crypts derived from PPARα-deficient mice were defective in organoid formation.^28^ In addition, PPARδ is implicated in regulating asymmetric division of hematopoietic stem cell.^29^ Given that MG53-TG intestine had increased expression of PPARα, we speculated that the effects of MG53 on ISCs may be mediated by PPARα. Therefore, we treated the organoids with a PPARα antagonist GW6471. Incubation with GW6471 largely blocked the asymmetric division induced by MG53 (Fig. 4a, b). Moreover, using *Notch1*and *Atoh1* as markers to distinguish absorptive and secretory lineages,^30,31^ respectively, organoids derived from MG53;Lgr5 mice exhibited a greater population of Atoh1^+^ cells and a reduced number of Notch1^+^ cells, which was abolished by GW6471 treatment (Fig. 4c). Consistently, GW6471 treatment eliminated the differences in the length of TA zone as well as the numbers of goblet cells between MG53;Lgr5 and Lgr5 mice in both the ileum and colon (Fig. 4d–f), highlighting that PPARα activation is critical for MG53-triggered biased ISC differentiation.

### Activation of PPARα signaling in ISC is required for MG53 to alleviate intestinal injury

**Fig. 5:**
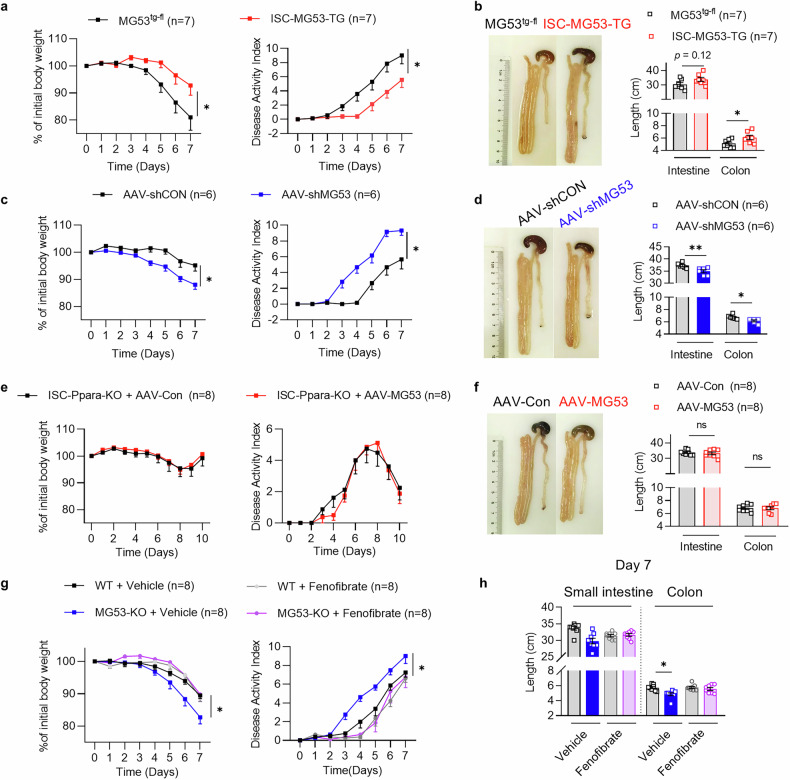
Activation of PPARα signaling is required for MG53-induced alleviation of intestinal injury. Body weight change and DAI (**a**) as well as the representative images and statistic results of the intestinal length (**b**) of ISC-MG53-TG and the control MG53^tg-fl^ mice. *n* = 7 for each group. Body weight change and DAI (**c**) as well as the representative images and statistic results of the intestinal length (**d**) of the Lgr5 mice with ISC-specific inhibition of MG53 expression via infection with adeno-associated virus with inducible expression of shRNA targeting MG53 (AAV-shMG53) or the control mice infected with control shRNA (AAV-shCON). *n* = 6 for each group. Body weight change and DAI (**e**) as well as the intestinal length (**f**) of ISC-Ppara-KO mice with or without ISC-specific MG53 overexpression via injecting AAV-MG53 or AAV-CON. *n* = 8 for each group. Body weight change and DAI (**g**) as well as the intestinal length (**h**) of MG53-KO and control WT mice treated with PPARα agonist fenofibrate or vehicle following DSS treatment. *n* = 8 for each group. Normal distribution was confirmed by Shapiro–Wilk test. Data were analyzed using two-tailed paired *t* test (**a**–**e**, **g**), Mann–Whitney *U* test (**f**, **h**), and were presented as mean ± s.e.m. ns not significant, *p < 0.05 and **p < 0.01 as compared with the corresponding controls

We went on to evaluate the function of MG53-PPARα axis in vivo. RNAscope in situ hybridization confirmed that endogenous MG53 is presented in ISCs (Supplementary Fig. 8a). We then determine the effects of MG53 in ISCs by crossing MG53^tg-fl^ with Lgr5-creERT2 mice to enable MG53 overexpression in Lgr5^+^ ISCs (ISC-MG53-TG). After challenging with DSS, the manifestations of intestinal injury were significantly mitigated in the ISC-MG53-TG mice (Fig. 5a, b and Supplementary Fig. 8b). To the contrary, when the Lgr5-creERT2 mice infected with adeno-associated virus pAAV-CBG-DIO-EGFP-miR30shRNA(Trim72)-WPRE (AAV-shMG53) to inhibit MG53 expression specifically in ISCs, they experienced more severe damage compared with those injected with the control virus pAAV-CBG-DIO-EGFP-miR30shRNA(NC)-WPRE (AAV-shCON) (Fig. 5c, d and Supplementary Fig. 8c). These observations indicated that ISC-specific overexpression of MG53 is sufficient to protect against intestinal injury.

To evaluate the function of PPARα in mediating the protective effect of MG53 against intestinal damage, mice were subjected to 2.5% DSS treatment in the presence or absence of GW6471 for 7 days (Supplementary Fig. 9a). The increased Lyz^+^ Paneth cell population was eliminated by GW6471 treatment (Supplementary Fig. 9b). Moreover, GW6471 blocked MG53 overexpression-induced protective effects in all aspects, including the body weight, DAI, as well as the colon length and the intestinal morphology (Supplementary Fig. 9c–e). Furthermore, we generated ISC-specific PPARα knockout mice (ISC-Ppara-KO) by crossing Ppara-floxed mice (Ppara^fl/fl^) with Lgr5-CreERT2 mice, and MG53 expression was introduced by injection of adeno-associated virus containing Cre-inducible expression vector of MG53 (AAV-MG53). Thus, treatment with tamoxifen induced the depletion of PPARα as well as overexpression of MG53 (Supplementary Fig. 9f) in the cells with CreERT2 expression. Remarkably, PPARα deficiency in ISCs fully blocked the beneficial effects of MG53 on DSS-treated mice (Fig. 5e, f). In terms of inflammatory responses, ISC-MG53-TG mice exhibited a significantly reduced CD45^+^CD3^+^CD8^+^ cell population in the intestine following DSS treatment as compared with the control MG53^tg-fl^ mice. To delineate the role of PPARα, we crossed the ISC-MG53-TG with Ppara^fl/fl^ mice (ISC-MG53TG-PparaKO) to allow ISC-specific *Ppara* deficiency as well as MG53 overexpression. Ablation of *Ppara* in ISC-MG53TG-PparaKO mice markedly blunted the reduction of CD45^+^CD3^+^CD8^+^ cell population caused by MG53 overexpression and increased the proportion of CD45^+^CD3^+^CD4^+^ cells. These results corroborate the function of MG53-PPARα in alleviating intestinal inflammation induced by DSS (Supplementary Fig. 9g).

In contrast, treatment of MG53-KO mice with a PPARα agonist, fenofibrate, was sufficient to improve the condition after DSS treatment, manifested by better maintained body weight, reduced DAI, and attenuated colon length shortening relative to vehicle-treated mice (Fig. 5g, h and Supplementary Fig. 9h–j). The results from these animal models demonstrate that MG53-induced upregulation of PPARα in ISCs is indispensable for MG53-induced protective responses against intestinal injury caused by DSS.

### PPARα activation by an endogenous ligand palmitoleic acid ameliorates IBD

**Fig. 6:**
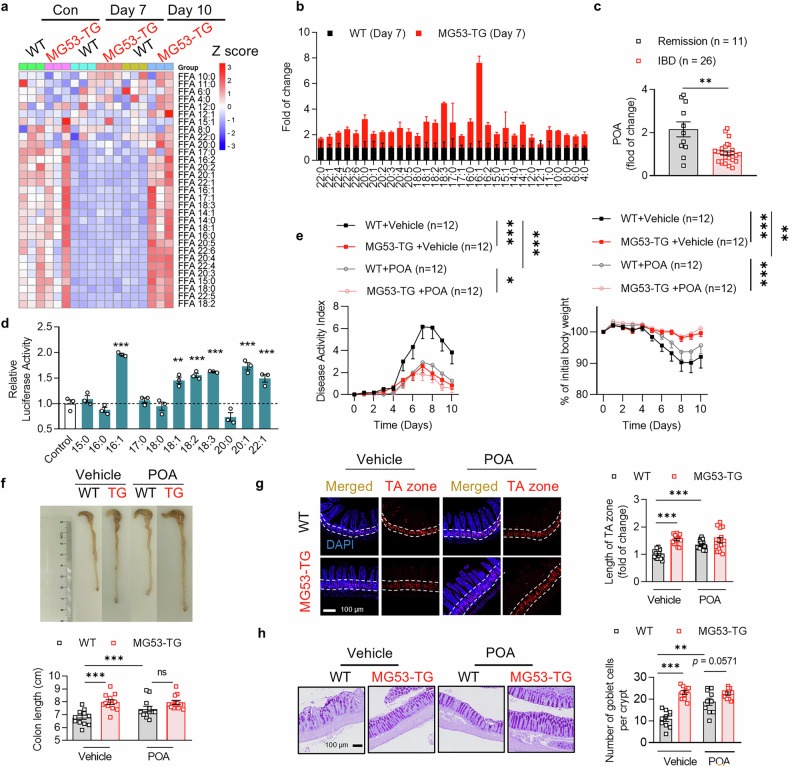
Enhanced PPARα activation by palmitoleic acid contributes to MG53-mediated amelioration of intestinal injury. **a** Heatmap of free fatty acids (FFA) in MG53-TG and WT intestinal tissues at the indicated time points during DSS treatment. *n* = 3 for each group. **b** Changes in the intestinal content of FFA in the MG53-TG and their WT littermates at day 7 after DSS treatment. *n* = 3 for each group. **c** Relative change in POA concentrations in the serum of IBD patients in remission (*n* = 11) or flare (*n* = 26). **d** Luciferase reporter activity driven by the PPARα-responsive element in the presence of different FFA in HEK293 cells. *n* = 3 for each group. Body weight change and DAI (**e**) as well as the colon length (**f**) of MG53-TG (TG) and their WT littermates challenged with DSS with or without POA treatment. *n* = 12 for each group. **g** Immunofluorescence staining of Ki-67 (red) and statistic results of the length of TA zone of MG53-TG and their WT littermates with or without POA treatment. *n* = 15 for each group. The nuclei were indicated by DAPI staining (blue); scale bar, 100 μm. **h** Representative images and statistic results of PAS staining of goblet cells in the colon after POA treatment. *n* = 10 for each group. Scale bar, 100 μm. Normal distribution was confirmed by Shapiro–Wilk test. Data were analyzed using one-way ANOVA with Tukey post hoc test (**c**, **e**), two tailed *t* test (**d**, **f**–**h**) and were presented as mean ± s.e.m. ns not significant, *p < 0.05, **p < 0.01, and ***p < 0.001 as compared with the corresponding controls

Since data from in vivo and the crypt organoids revealed that upregulation of MG53 elevated the expression of genes regulating PPAR signaling and fatty acid metabolism (Fig. 3b–d), we then sought to explore the potential relationship between the alterations in fatty acid metabolism and activation of PPARα which facilitates the protective effects of MG53. To this end, we employed GC/MS-based metabolomics to detect free fatty acids (FFA) in MG53-TG mice during the progression or remission of DSS-induced intestinal injury. Sharp decline in FFA levels was found in the distal ileum of both MG53-TG and WT mice following DSS treatment. Nevertheless, upregulation of MG53 enabled a rapid recovery of FFA after a healing phase (day 10), associated with faster and better tissue repair in the MG53-TG mice (Fig. 6a). We then focused on the changes in FFA during the progression of intestinal injury right after the 7-d DSS treatment (Supplementary Fig. 10a). The contents of FFA examined were all greater in the MG53-TG. In particular, the level of palmitoleic acid (POA, C16:1n-7) was increased to more than 7-fold of that in the WT littermates (Fig. 6b). It is noteworthy that circulating POA level was lower in the patients with IBD (Supplementary Fig. 10b, c). Furthermore, compared to patients in remission, individuals experiencing flare of IBD demonstrated lower serum POA levels (Fig. 6c and Supplementary Table S1). MG53-TG mice exhibited increased expression of intestinal fatty acid transporters (Supplementary Fig. 10d). Moreover, MG53 overexpression promotes POA uptake by HIEC-6 via inducing the expression of fatty acid transporters (Supplementary Fig. 10e, f). Importantly, we found that these unsaturated fatty acids, especially POA, were potent agonists of PPARα, as assessed by luciferase activity of PPARα-responsive reporter in HEK293T cells (Fig. 6d). Functionally, POA treatment almost phenocopied MG53 overexpression in response to DSS challenge, except for limited effect on body weight change (Fig. 6e, f and Supplementary Fig. 10g, h). In particular, the elongation of TA zone and enhanced secretory lineage commitment of ISCs were also observed in the POA-treated mice (Fig. 6g, h and Supplementary Fig. 10i). Taken together, MG53 upregulates PPARα expression as well as increases the content of unsaturated fatty acids including POA that act as agonists for PPARα, thus reducing susceptibility to intestinal injury via regulating ISC cell fate by PPARα activation.

## Discussion

Inflammation is a major clinical manifestation of IBD and current treatments have primarily focused on targeting inflammatory pathways. However, these approaches carry risk of repressing immune responses and increasing susceptibility to infections. Moreover, anti-inflammation therapies fail to address the core issue of restoring intestinal epithelial integrity. Recently, several studies have demonstrated the essential role of ISCs in maintaining epithelial homeostasis and their potential as therapeutic targets. In this study, we found that MG53 promotes the proliferation and differentiation of ISCs, which in turn, ameliorates intestinal damage and the ensuing inflammation.

We have shown that MG53 overexpression attenuated acute IBD triggered by DSS or LPS, whereas MG53 deficiency aggravated these insulting stimuli-induced intestinal injuries. Overexpression of MG53 significantly reduced inflammatory cytokine levels in circulation as well as in the intestinal tissues, indicating mitigated inflammation in MG53-TG mice. Importantly, MG53-TG mice displayed an elongated TA zone and accelerated turnover of intestinal epithelial cells, suggesting the beneficial effects of MG53 are associated with ISCs. Indeed, increasing the expression of MG53 in ISCs was sufficient to protect intestinal tract against various injuries. In contrast, ISC-specific ablation of MG53 aggravated the intestinal damage. However, the population of Lgr5^+^ stem cells was decreased, while the proportion of the secretory progenitors was increased in MG53-TG intestine. This is because that MG53 facilitates the asymmetric division of ISCs biased towards secretory cell lineages by activating PPARα signaling. Specifically, MG53 not only upregulated PPARα at the transcriptional level but also enhanced its activity by increasing endogenous ligands, a panel of unsaturated fatty acids, such as POA, in the intestine (Supplementary Fig. 11). In contrast, PPARα depletion or inhibition fully abrogated MG53-mediated biased differentiation of ISCs and the resultant intestinal protection. Remarkably, administration of POA, similar to the upregulation of MG53, elicited beneficial effects in mice subjected to harmful insults, corroborating the critical role of PPARα in mediating the protective function of MG53 in the gut. Our findings reveal MG53-PPARα as important targets and POA as a therapeutic agent for IBD treatment.

The rapid replenishment of damaged epithelial cells by enhanced differentiation of progenitor cells had an essential contribution to the MG53-induced alleviation of intestinal damage, whereas inhibiting the expression of MG53 aggravated the injuries. Remarkably, enhancing MG53 expression favors asymmetric division with biased commitment to secretory lineage at the expense of reduced stem cell population, as manifested by fewer Lgr5^+^ cells, but longer TA zone with more progenitors and matured secretory cells in the MG53-TG mice. This screwed cell type composition of ISC niche may prime the MG53-TG intestine into a more active state of replacement and repair against various insults. In this regard, it has been shown that the secretory progenitors exhibit high plasticity by dedifferentiating into stem cells upon damage.^9,10,32^ The dedifferentiation capacity is also observed in both mature and committed secretory cells in the intestine^33^ and the lung^32^. In addition to enhancing regeneration competency, increased secretory cell population can produce more mucin to form mucosal surface against external insults.^34^ Thus, disrupting the intricate balance of ISC dynamics may contribute to inflammatory disorders and the pathogenesis of colitis-associated cancers.^35^

Mechanistically, we have shown that the beneficial effects of MG53 rely on the transcriptional upregulation of PPARα. Thus, PPARα activation is required and sufficient to mediate MG53-induced protection of intestinal epithelial integrity. Furthermore, MG53-PPARα axis ameliorates intestinal damage via promoting ISC secretory lineage commitment and fatty acid uptake. While no genetic mutations in MG53 or PPARα has been implicated in the pathogenesis of IBD, impaired PPARα signaling has been linked to the pathogenesis of colitis^23^ as well as AOM or AOM/DSS-induced colon carcinogenesis^36^. Moreover, we have identified a panel of unsaturated fatty acids, exemplified by POA, as PPARα agonists to elicit potent therapeutic effects in the mouse model of IBD. As such, POA may be used as a safe and effective therapeutic agent. In addition, consuming foods with high POA content, such as salmon and macadamia nuts, may provide an effective and convenient means of preventing and treating IBD syndrome. Interestingly, fenofibrate introduced via intraperitoneal injection was shown to worsen the outcome of intestinal injury.^37^ The discrepancy might be caused by the route of administration. Intraperitoneally injected fenofibrate was predominantly absorbed by hepatocytes. Activation of PPARα in the liver enhances the release of pigment epithelium-derived factor, which in turn, restrained ISC proliferation and thereby exacerbated intestinal damage. In our study, fenofibrate was administered via oral gavage, and was taken up mainly by intestinal cells, instead of the liver. Our observation was corroborated by other studies using oral administration of fenofibrate, which also reported its beneficial effects in ameliorating AOM/DSS-induced inflammation in the colon.^36^

In addition to activating PPARα signaling, we cannot fully exclude other potential mechanisms contributing to the protective effects of MG53. For instance, MG53 is originally reported as a key regulator of membrane repair that preserves cell integrity in multiple organs upon acute injury.^17^ Indeed, MG53 overexpression markedly reduced DSS-induced apoptotic cell death, suggesting that the function of MG53 in membrane repair may be involved. In addition, our recent work has established MG53 as an E3 ligase of cyclin D and repressing the growth of colorectal tumors by promoting cyclin D1 degradation.^20^ Though our preliminary data suggest that the protective function of MG53 in IBD does not require E3 ligase activity, further investigation will determine whether the E3 ligase activity and membrane repair function contribute to the MG53-mediated protection against IBD.

In summary, MG53 plays a potent protective role in intestine, especially under pathophysiological stress. MG53 promotes secretory lineage commitment by facilitating the asymmetric division of ISCs in a PPARα-dependent manner, minimizing inflammatory damage and enhancing lipid metabolism. These findings underscore the importance of targeting MG53-PPARα signaling for the treatment of IBD.

## Materials and methods

### Human biological material

Patient samples were obtained from Peking Union Medical College Hospital, Beijing, China. The detailed information is provided in Supplementary Table S1. All the donors were informed about the study and provided consent prior to sample collection. The procedures approved by the Human Ethics Committees of Peking Union Medical College Hospital (I-24PJ0139) were performed in compliance with standards of research involving human subjects.

### Mice

Animals were housed in the Laboratory Animal Center at Peking University, Beijing, China, under a standard 12-h light/12-h dark cycle, with water and food provided ad libitum. The animal facility is accredited by the Association for Assessment and Accreditation of Laboratory Animal Care and the animal protocols were approved by the Institutional Animal Care and Use Committee of Peking University. The allocation of animals into experimental groups was randomized.

The construction and characteristics of MG53-TG and MG53-KO mice were reported previously.^14,38,39^ Lgr5-creERT2 mice were from Jackson Lab (#008875). Ppara^fl/fl^ mice were purchased from Cyagen (S-CKO-04396). MG53^tg-fl^ was generated by our laboratory.

### Mouse models

#### DSS treatment

A total of 2.5% (w/v) DSS was provided in the drinking water. Mice were subjected to DSS water for 7 days, and switched to normal drinking water for another 3 days. The body weights and disease activity indexes (DAI) were monitored throughout the experiment. The DAI is a composite score that takes into account of weight loss, stool consistency, and rectal bleeding. Specifically, the scores were determined as follows: *body weight loss* (% of baseline): 0: unchanged, 1: 1–5%, 2: 6–10%, 3: 11–20%, and 4: greater than 20%; *stool consistency*: 0: normal, 2: mild loose stool, 4: diarrhea; *rectal bleeding*: 0: none, 2: hemoccult positive stools, 3: visible blood on stool, and 4: gross bleeding per rectum. Mice were euthanized at the indicated time points, and small intestines and colons were collected immediately. For histological examination, small intestine and colon specimens were embedded in paraffin following fixation in 4% paraformaldehyde (PFA).

#### Lipopolysaccharide (LPS) treatment

Mice were intraperitoneally injected once with LPS dissolved in saline at 50 mg kg^−1^. To examine the involvement of microbiota, mice were administered with antibiotic cocktail (1 g ampicillin, 0.5 g Vancomycin, 1 g Metronidazole, 1 g Neomycin, and 10 g sucrose dissolved in 1 L water) for 4 weeks.

#### Irradiation

For γ irradiation, mice were anesthetized and placed in the RS2000-160KeV irradiator, and adjusted the instrument to the specified irradiation intensity.

#### Adeno-associated virus (AAV) and tamoxifen treatment

For inducible MG53 overexpression in ISCs, AAV-EF1α-DIO-MG53-mCherry-WPRE (AAV-MG53, OBiO Technology) and control virus AAV-EF1α-DIO-mCherry-WPRE (AAV-CON) were injected intravenously at a titer of 10^11^ Vg per mouse. For knocking down the expression of MG53, pAAV-CBG-DIO-EGFP-miR30shRNA(Trim72)-WPRE (AAV-shMG53) and control virus pAAV-CBG-DIO-EGFP-miR30shRNA(NC)-WPRE (AAV-shCON) were injected. Six weeks after injection, mice received intraperitoneal administration of 100 μl Tamoxifen (Sigma, 10540-29-1) suspended in corn oil (Macklin, C805618) at concentration of 20 mg mL^−1^.

#### Treatment with drugs

γ irradiation was performed using irradiator RS2000-160keV (Rad Source Technologies Inc.). GW6471 was dissolved in 10% DMSO, 40% PEG-300, 5% Tween-80, and 45% saline and orally administered at 10 mg kg^−1^ every day for 7 days. Fenofibrate was prepared in corn oil at 10 mg mL^−1^ and sonicated until the liquid is clear. Mice were treated with fenofibrate by oral gavage at 100 mg kg^−1^ every day. Palmitoleic acid (POA, Sigma, P9417) was dissolved in 1% free fatty acids-free BSA and given to mice by oral gavage at 300 mg kg^−1^ every day for 7 days.

### Organoid isolation and cell culture

#### Intestinal organoids

Small intestines were surgically removed, opened longitudinally and flushed with cold PBS as previously described.^40,41^ Then, small pieces of intestine (~5 mm) was incubated with 5 mM EDTA-PBS at 4 °C for 30 min. Following the incubation, the intestine was transferred to PBS and agitated vigorously. The supernatant was filtered through 100-μm filter. The collection step was repeated 3 times. The combined cell suspension was filtered through 70-μm strainers and centrifuged at 300 × *g* for 5 min. The isolated crypts were embedded in Matrigel (Corning, 356231) at a density of approximately 100 crypts per μl and cultured in IntestiCult OGM medium (STEM CELL, 06005). The culture medium was replenished every 2 days, and the organoids were passaged every 5 days. To passage, the organoids were incubated with Dissociation Reagent (STEM CELL, 0485) at 4 °C for 10 min, and then disaggregated by pipetting for 50 times. The organoids were then washed 3 times with cold PBS and re-embedded in Matrigel for further culture.

For imaging of organoids, the Matrigel was plated in glass-bottom 24-well plate (Eppendorf, 30741021). Images were captured using Nikon A1RSi+ laser scanning microscope (Nikon). For FACS analysis, organoids digested with TrypLE Express (Gibco, 12605010) were filtered through strainers, and resuspended in 2% BSA (PBS). Then cells were incubated with primary antibodies for 30 min at 4 °C, and then washed with 2% BSA in PBS for 3 times. Subsequently, the cells were incubated with secondary antibodies at 4 °C for 30 min and washed again. Before flow cytometry analysis, 7-AAD (BD Biosciences, 559925) was added to the cells. The FACS was performed using Aria SORP (BD Biosciences).

For pair-cell assay, organoids were digested into single cells. Lgr5-positive doublets were isolated using flow cytometry and plated in glass-bottom 24-well plate (Eppendorf, 30741021). For immunofluorescence staining, PFA-fixed cells were washed with immunofluorescence staining (IF) buffer (0.2% Triton X-100, 0.05% Tween in PBS) twice, and permeabilized with 0.5% Triton X-100 (in PBS) for 20 min. After two additional washes with IF buffer, cells were incubated in the blocking buffer (1% BSA in IF buffer) for 30 min and then switch to fresh blocking buffer containing anti-GFP (CST, 55494S) and anti-alpha-Tubulin (HUABIO, ER130905), and then incubated at 4 °C overnight. The next day after washing, the incubation with secondary antibodies was for 1 h at room temperature in the dark. Following final washing, the cells were mounted with mounting solution with DAPI.

#### Colonic organoids

Colons were surgically removed, opened longitudinally and flushed with cold PBS. Subsequently, the colon tissue in ~2 mm pieces was incubated in 2 mM EDTA-PBS at 4 °C for 30 min. Following the incubation, the colon was transferred to PBS and agitated vigorously. The supernatant was filtered through 70-μm filter and centrifuged at 300 × *g* for 5 min. The isolated crypts were embedded in Matrigel (Corning, 356231) at a density of approximately 100 crypts per μl and cultured in IntestiCult OGM medium (BioGenous, K2204-MC). The culture medium was replenished every other day, and the organoids were passaged every 5 days. To subculture, the medium was removed and replaced with cold PBS. The organoids were then incubated with organoid dissociation solution (BioGenous, E238001) at 37 °C for 1 min, centrifuged at 300 × *g* for 5 min, washed 3 times with cold PBS, and re-embedded in Matrigel for further culture.

#### Human intestinal organoids

The human intestinal organoids (BioGenous, HL-OL002) were culture according to manufacturer’s instructions. After thawing, the organoids were planted in Matrigel and cultured in Human Intestinal Organoid Kit (BioGenous, K2002-HI-OL002). The medium was replaced every 2–3 days. For organoids passage, the organoids were suspended in Organoid Dissociation Solution (BioGenous, E238001) for 2 min at room temperature followed by pipetting for 50 times. The organoids were then washed re-embedded in Matrigel for further culture. For further treatment, the organoids treated with 40 ng mL^−1^ TNF-α (Novoprotein, C008), with or without fenofibrate (40 μg mL^−1^, dissolved in DMSO) or palmitoleic acid (POA) (40 μg mL^−1^, dissolved in 1% BSA in PBS).

### scRNA-seq: tissue dissociation, cell isolation, library preparation, and sequencing

Crypts isolated from mouse intestine were incubated with TrypLE Express (Gibco, 12605010) at 37 °C for 60 s. The cell suspension was passed through a 40-μm filter, then stained with EpCAM (Thermo Fisher, 14-5791-81) and 7-AAD (BD Biosciences, 559925), and sorted by FACS to enrich 7-AAD^−^EpCAM^+^ intestinal epithelial cells.

Single-cell RNA sequencing (scRNA-seq) libraries were constructed using the 10 × Genomics single cell 3′ Library and Gel Bead Kit V3 (10 × Genomics, 1000075) following the manufacturer’s instructions. Reverse transcription was performed at 53 °C for 45 min and switched to 85 °C for 5 min to terminate reaction. The cDNA library was generated using the Agilent 4200. The sequencing was carried out on the Illumina Novaseq6000 sequencer. The estimated number of cells is 27,513, fraction reads in cells is 92.3%, median genes per cell is 1366, and the median UMI counts per cell is 5159. Quality control was performed to remove low-quality cells based on the following criteria: (1) <200 gene expressed; (2) <99 unique molecular identifiers (UMIs); (3) >25% UMIs derived from mitochondrial genome. Subsequent analysis was performed using the R package Seurat. Cell clusters were annotated based upon the expression of canonical marker genes. Pseudotime calculations were conducted using Monocle 2, with stem cell as the origin of differentiation. ‘AddModuleScore’ function implanted in Seurat R package was utilized to perform calculation of signature scores. Genes included in the calculation of scores for stem cells were *Lgr5*, *Ube3c*, *Mki67*, and *Myc*; genes for absorptive lineage were *Arg2*, *Apoa4*, *Aldob,* and *Hes1*; genes for secretory cells were *Dll1*, *Muc2*, *Ang4*, and *Pou2f3*.

### Immunohistochemistry and immunofluorescence staining

Small intestine and colon tissues were washed with cold Ringer’s buffer (25 mM NaHCO_3_, 115 mM NaCl, 1.2 mM MgCl_2_, 1.2 mM CaCl_2_, 2.4 mM K_2_HPO_4_, 0.4 mM KH_2_PO_4_, pH 7.3), fixed in 4% PFA, and embedded in paraffin.

#### Immunofluorescence staining

Paraffin-embedded intestinal sections were dewaxed in xylene and rehydrated. The slides were then subjected to antigen retrieval buffer (ZSGB-BIO, ZLI9071) by heating in microwave oven for 10 min. Tissue sections were blocked in 10% goat serum (ZSGB-BIO, ZLI9021) in PBS. Incubation with primary antibodies anti-Lysozyme (1:500, BOSTER, BM4383), anti-Ki-67 (1:500, CST, 9129S), anti-PPARα (1:500, Santa Cruz Biotechnology, sc-398394), or anti-BrdU (1:250, Santa Cruz Biotechnology, sc-32323) was at 4 °C overnight and then washed with PBST (0.4% Tween-20). Incubation with anti-rabbit or anti-mouse IgG antibody (1:250; ZSGB-BIO, ZF-0321, ZF-0311, ZF-0513, and ZF-0516) were at room temperature for 60 min. Tissue sections were then mounted with mounting medium containing DAPI (ZSGB-BIO, ZLI-9557).

#### TdT-mediated dUTP nick-end labeling (TUNEL) assay

TUNEL assay was carried out using in situ cell death detection kit (Roche, 12156792910) as described by the manufacturer. Samples were analyzed under fluorescence microscope with excitation wavelength of 450–500 nm and detection wavelength of 515–565 nm.

#### Periodic acid-Schiff (PAS) staining

Tissue sections were stained with PAS Stain Kit (Solarbio, G1281) according to manufacturer’s protocol.

#### Tissue array

The array contains human small intestine samples (US Biomax, SM802) and human colon samples (US Biomax, CO245b). Samples were labeled with fluorescence using NEON 5-color Allround Discovery Kit for FFPE (Histova. Biotechnology, NEFP5100), and the fluorescent images were acquired using a Hamamatsu Photonics fluorescence microscope. The tissue array was analyzed using Halo platform (Version 3.3).

### BrdU/EdU dual-labeling

BrdU (100 mg kg^−1^, Millipore Sigma, B5002) and EdU (10 mg kg^−1^, Millipore Sigma, 900584) were dissolved in sterile PBS. BrdU was intraperitoneally injected 48 h before euthanasia, and EdU was injected 24 h before euthanasia. Tissue samples were fixed in formalin and embedded in paraffin. BrdU was detected using anti-BrdU (Santa Cruz Biotechnology sc-32323) and EdU was detected using the ClickiT Plus EdU Alexa Fluor 647 Imaging Assay Kit (Thermo Fisher Scientific, C10340).

### RNAscope in situ hybridization

The RNAscope LS multiplex Fluorescent Reagent Kit (ACD, 322800) was employed following the standard protocol by manufacturer. Specific probes for Lgr5 (ACD, 312171) and the customized MG53 probe were purchased from ACD.

### Cell transfection and luciferase reporter assay

To knockdown MG53 expression in HCT116, cells were infected with lentivirus (Suzhou GenePharma) expressing shRNA specifically targeting MG53 (sequence of shRNA: 5’-GACTGAGTTCCTCATGAAATA-3’), as previously described.^20^

HIEC-6 cells were infected with adenovirus expressing either MG53 or β-gal (Beijing BAC Biological Technologies) as previously described.^20^ Twenty-four hours after infection, cells were collected.

HIEC-6 cells were co-transfected with reporter constructs driven by nested deletion of PPARα promote, pRL-TK plasmid, and expression vector pcDNA4-MG53 using Lipofectamine 3000 (Invitrogen, L3000001). The cells were harvested 48 h post-transfection and luciferase activity was assessed in cell lysates using the Firefly Luciferase Assay Kit (Tiosbio, F6024) following the manufacturer’s instructions.

### POA uptake assay

HIEC-6 cells were infected with adenovirus (30 MOI) expressing MG53 or β-gal as a control. Twenty-four hours after infection, cells were incubated with 100 μM POA (in 1% free fatty acid-free BSA) for 5 min before harvesting. Cell lysate was collected for assessing POA content by mass spectrometry.

### RNA extraction and RT-qPCR

Total RNA was prepared using TRIzol reagent (Invitrogen, 15596026) and reverse transcribed using PrimeScript RT reagent Kit (Takara, RR047A). Real-time PCR was performed with HiScript II Q RT SuperMix (Vazyme, R222-01) on LightCycler 96 (Roche).

### Cytokines ELISA

Serum was collected from mice and incubated at room temperature for 30 min, followed by centrifugation at 3000 rpm for 5 min at 4 °C. The levels of IL-6 and IL-18 were assessed using Mouse ProcartaPlex Kit (Invitrogen) and analyzed using the Luminex 200 platform.

### RNAseq

RNA was extracted from freshly collected mouse intestine tissues or organoids. The library construction was performed by Genewiz, Inc., Beijing. Sequencing was performed on an Illumina platform and reads were assessed for quality and trimmed for adapter sequences using FastQC (version 0.10.1) and Cutadapt (version 1.9.1). The clean reads were mapped to the mouse reference genome by Hisat2 (version 2.0.1). The FPKM (Fragments Per Kilobase of transcript per Million mapped fragments) was normalized and concerted by Heseq (version 0.6.1). Differential genes were determined with a significance cutoff of *q*-value ≤ 0.05.

### Metabolomics of free fatty acids

Freshly obtained intestine tissues were extracted with 80% methanol and stored at −80 °C. Metabolomics analysis was carried out with MRM (Multiple Reaction Monitoring mode) mode of UPLC-QQQ-MS/MS to quantify free fatty acids via positive and negative ionization. Samples were separated by an ACQUITY UPLC I-Class system (fixed loop), equipped with a CORTECS T3 2.7 μm (2.1*3.0 mm) analytical column. Raw data was processed with Progenesis QI software.

### Online statistical analysis

The Metascape online platform was used to perform Gene Set Enrichment Analysis (GSEA) and Kyoto Encyclopedia of Genes and Genomes (KEGG) pathways enrichment. *p* < 0.01 was applied as significance threshold, and the results were visualized using the online website (http://www.bioinformatics.com.cn/). POA in patient was analyzed using published data.^42^

### Statistical analysis

Statistical analysis was performed using GraphPad Prism 8.0.1 (GraphPad Software Inc., CA, USA). Normal distribution was evaluated using the Shapiro–Wilk test. For normally distributed data, analysis of 2-group samples utilized two-tailed Student *t* test or Welch *t* test (in cases of unequal variances), while >2-group analysis employed one-way ANOVA with post hoc Tukey multiple comparisons test. For non-normally distributed data or datasets with less than 6 observations, 2-group analysis was conducted using the Mann–Whitney *U* test, and >2-group analysis utilized the Kruskal–Wallis test with post hoc Dunn multiple comparisons test. Kaplan–Meier plots for survival were performed using the log-rank test. *p* < 0.05 was considered as statistically significant. No statistical method was used to predetermine sample size. Double-blind trials were taken during data collection and analysis.

## Supplementary information

20250516_MG53 in IBD_Supplementary information

20250430_MG53 in IBD_Raw WB and FACS gating info

## Data Availability

RNA-seq data and single-cell RNA sequencing dataset were deposited in the Gene Expression Omnibus (GEO). The accession numbers are GSE267687 and GSE295764, respectively. The metabolomics data are available under accession numbers: DOI: 10.6084/m9.figshare.29011070 and DOI: 10.6084/m9.figshare.29010560. All other data supporting the findings of this study are available from the corresponding authors upon reasonable request.
