# Supplementary material for: Mitsugumin 53 drives stem cell differentiation easing intestinal injury and inflammation
Source: Signal Transduct Target Ther. 2025 Jun 11;10:183. doi: 10.1038/s41392-025-02268-x (PMC12152187; doi:10.1038/s41392-025-02268-x)
Supplement: Supplementary file 1 — 20250516_MG53 in IBD_Supplementary information [file 41392_2025_2268_MOESM1_ESM.docx]

Supplementary Materials for

Mitsugumin 53 drives stem cell differentiation easing intestinal injury and inflammation

**Authors:** Yumeng Pei^†^, Meng Fang^†^, Hong-Kun Wu, Qionghua Cui, Li Quan, Xiaochuan Li, Keyi Zhang, Peng Xie, Peng Jiang, Yuan Liu, Meimei Huang, Fengxiang Lv, Xiaomin Hu, Ye-Guang Chen, Xinli Hu*, Rui-Ping Xiao*

Correspondence to***:*** [xiaor@pku.edu.cn](mailto:xiaor@pku.edu.cn); [huxxx025@pku.edu.cn](mailto:huxxx025@pku.edu.cn)

**This PDF file includes:**

Figures. S1 to S11

Table S1.

**
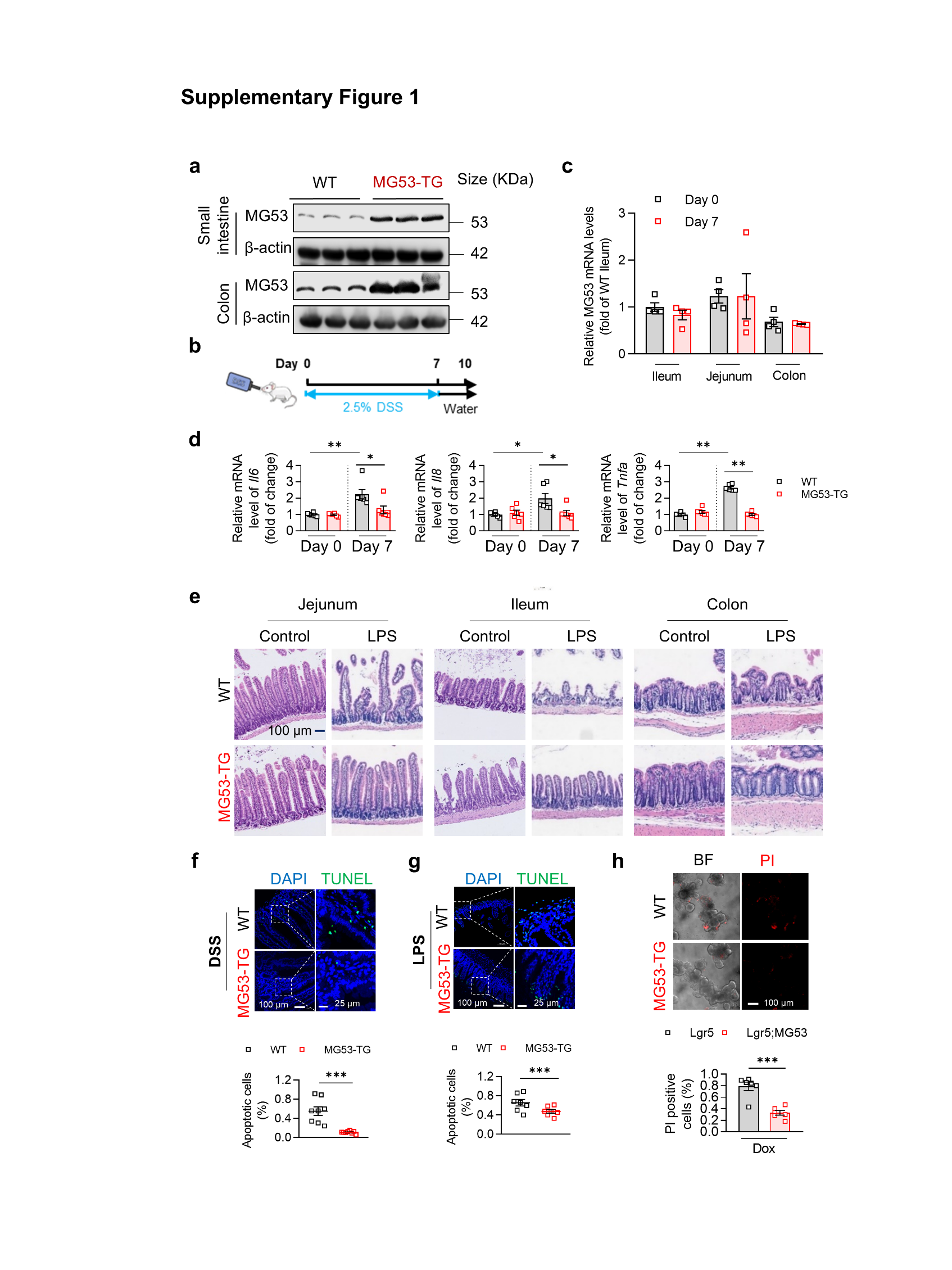
**

**Fig. S1.** MG53 protects mice against DSS-induced intestinal damage. **a** Western blots showing MG53 protein levels in the small intestine and colon of MG53-TG and WT mice. **b** Experimental design of DSS-induced IBD model. **c** Relative mRNA levels determined by RT and real-time PCR of MG53 in the ileum, jejunum, and colon on day 0 and day 7 after DSS treatment. *n* = 4 for each group. **d** Relative mRNA levels of IL-8, IL-6, and TNF-α in the WT and MG53-TG intestinal tissues after DSS treatment. *n* = 6 for each group. **e** Representative images of H&E staining of the jejunum, ileum, and colon of MG53-TG and WT mice injected with LPS or vehicle (Control). Scale bar, 100 μm. **f-g** Representative images of TUNEL staining and statistic results of apoptotic cells of the MG53-TG and WT intestinal tissues after challenged with DSS (**f**) or LPS (**g**). *n* = 8 for each group. Scale bars, 100 μm. **h** Representative images and statistical results of PI (red) positive cells after doxorubicin (DOX) treatment. *n* = 6 for each group. Scale bars, 100 μm.. Normal distribution was confirmed by Shapiro-Wilk test. Data were analyzed using *t* test (**c**, **d**, **f**, **g**, and **h**). All data were presented as mean ± s.e.m. * *P* < 0.05, ***P* < 0.01, and ****P* < 0.001 as compared with the corresponding controls.

**
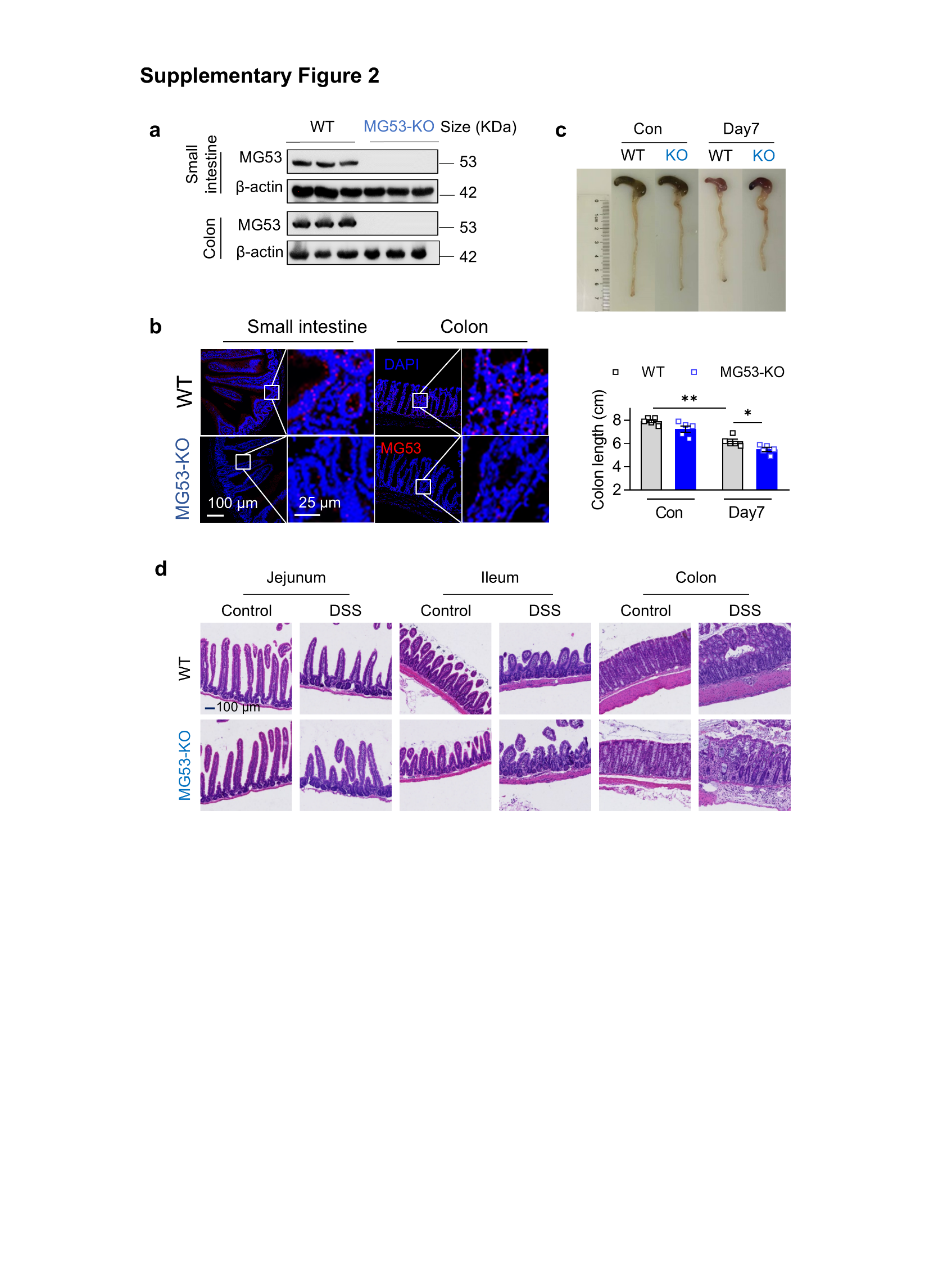
**

**Fig. S2.** MG53 deficiency aggravates DSS-induced intestinal injury in mice. **a** Western blots showing MG53 protein levels in the small intestine and colon of the MG53 knockout (MG53-KO) mice and their wild type littermates (WT). **b** Representative images of RNAscope showing MG53 (red) expression in the small intestine and colon of MG53-KO mice and their WT littermates. Nuclei were stained with DAPI (blue); scale bars, 100 μm. **c** Representative images and statistic results of the colon length of MG53-KO (KO) and WT mice on day 0 and day 7 of DSS treatment. *n* = 5 for each group. **d** Representative H&E staining images of the jejunum, ileum, and colon of MG53-KO and WT mice. Scale bar, 100 μm. Normal distribution was confirmed by Shapiro-Wilk test. In **b**, data were analyzed using Mann-Whitney *U* test and were presented as mean ± s.e.m. **P* < 0.05 and ***P* < 0.01 as compared with the corresponding controls.

**
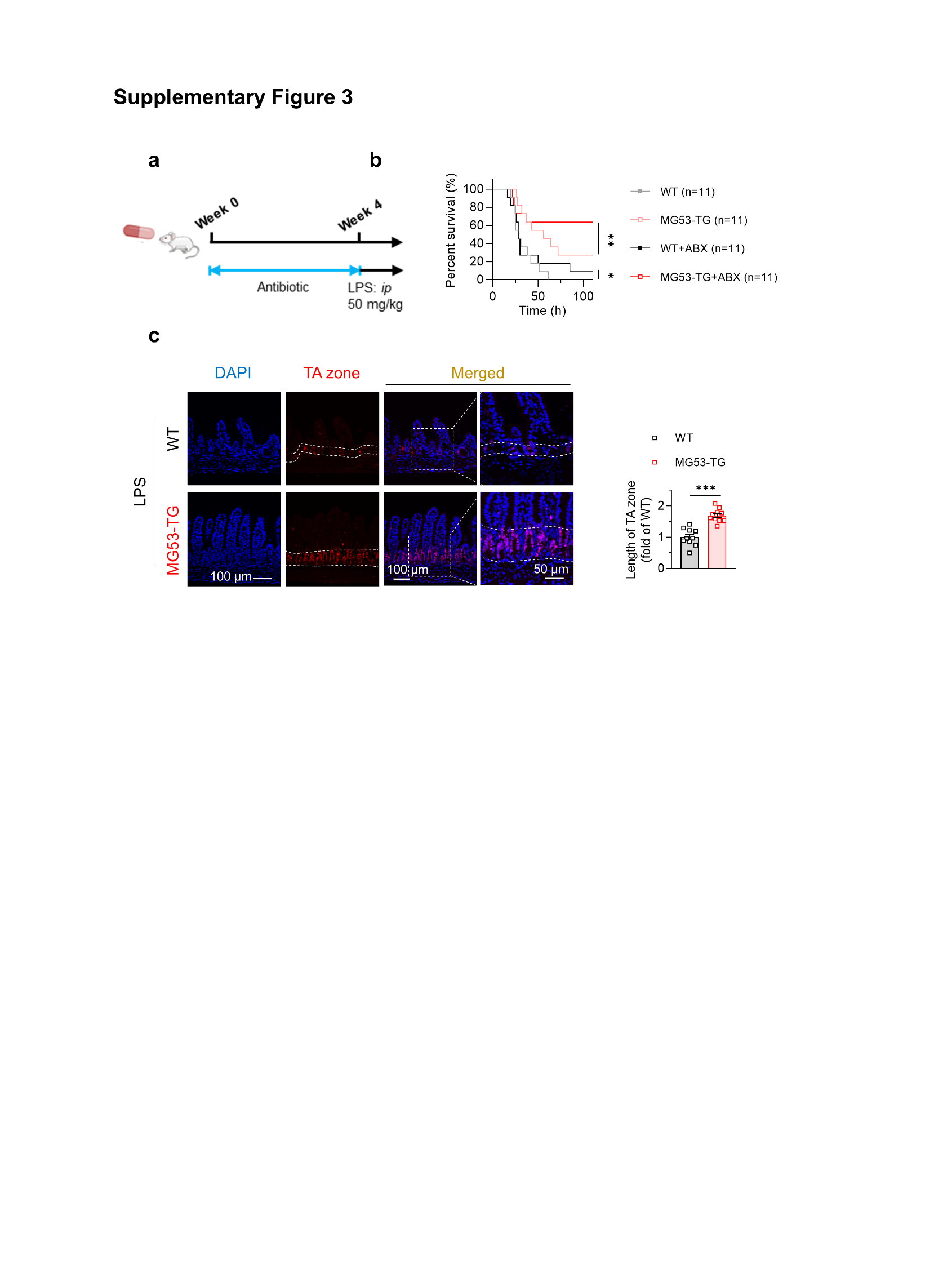
Fig. S3.** MG53 protects mice against LPS-induced intestinal damage. **a** Experimental design of LPS-induced intestinal damage. **b** Kaplan-Meier survival analysis comparing MG53-TG with WT after injected with 50 mg/kg LPS with or without antibiotic (ABX) treatment. *n* = 11 for each group. **c** Immunofluorescence staining of Ki-67 (red) and statistic results of the length of TA zone of MG53-TG and WT mice on day 3 after LPS treatment. *n* = 10 for each group. Nuclei were stained with DAPI (blue). Scale bars were as indicated. Normal distribution was confirmed by Shapiro-Wilk test. Data were analyzed using Mantel-Cox test (**b**) and *t* test (**c**). All data were presented as mean ± s.e.m. **P* < 0.05, ***P* < 0.01, and ****P* < 0.001 as compared with the corresponding controls.

**
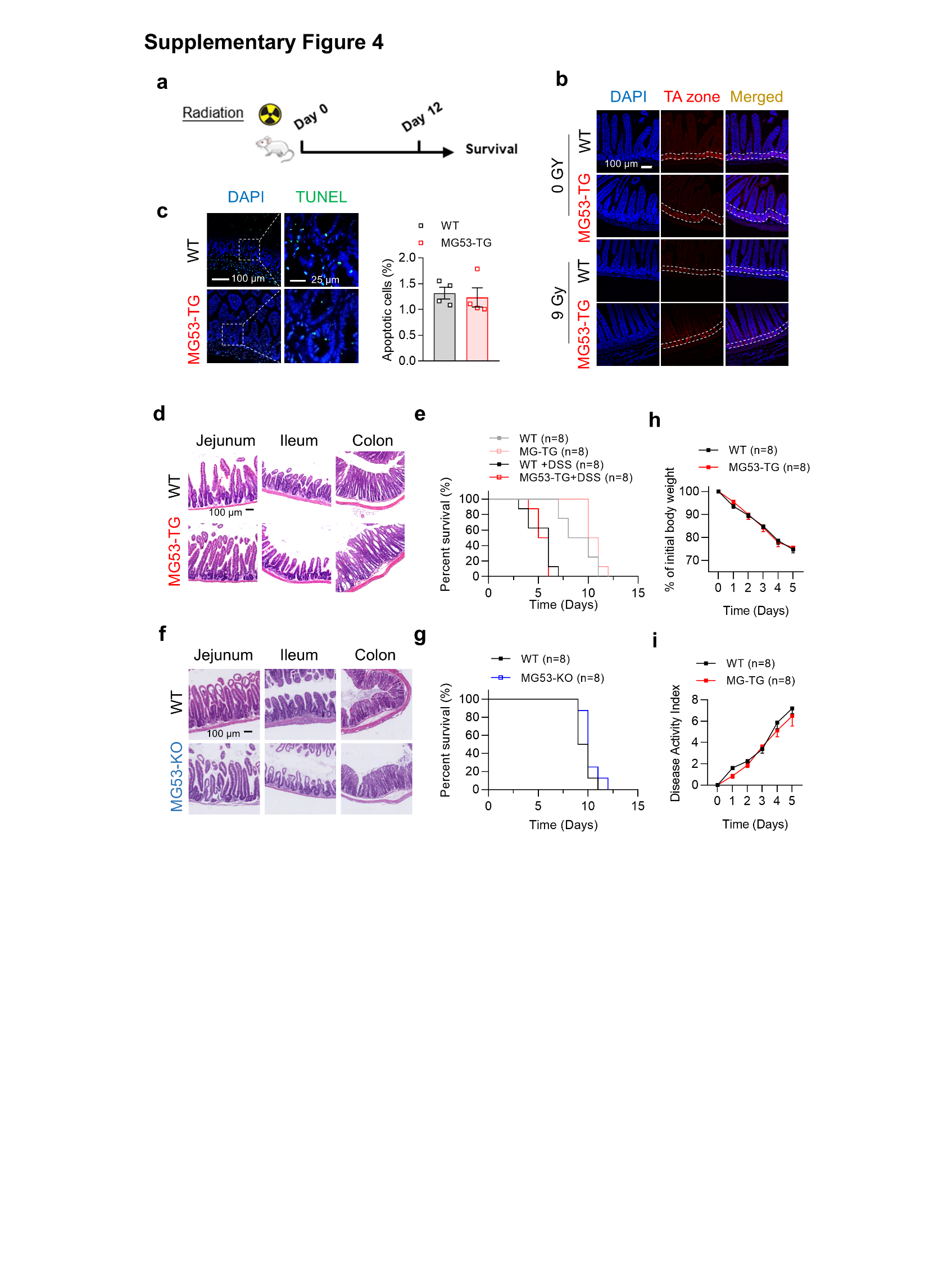
Fig. S4.** Transit amplifying (TA) zone is essential for MG53-mediated intestinal injury repair. **a** Experimental design of using γ irradiation to eliminate of TA zone. **b** Immunofluorescence staining of Ki-67 (red) to indicate the TA zone of MG53-TG and WT mice after γ irradiation at 9 Gy. Nuclei were stained with DAPI (blue); scale bar, 100 μm. **c** TUNEL staining and the statistic results of apoptotic cells in the intestine of MG53-TG and their WT littermates after γ irradiation. *n* = 4 for each group. Scale bar, 100 μm. **d** Representative H&E staining images of the jejunum, ileum, and colon of MG53-TG and WT controls after γ irradiation at 9 Gy. Scale bar, 100 μm. **e** Kaplan-Meier survival analysis of MG53-TG and their WT littermates after γ irradiation with or without DSS treatment. *n* = 8 for each group. **f** Representative H&E staining images of the jejunum, ileum, and colon of MG53-KO and their WT controls after γ irradiation at 9 Gy. Scale bar, 100 μm. **g** Kaplan-Meier survival analysis of MG53-KO and their WT littermates after γ irradiation. *n* = 8 for each group. **h-i** The body weight change (**h**) and DAI (**i**) of MG53-TG and WT mice after γ irradiation followed by DSS treatment. *n* = 8 for each group. Normal distribution was confirmed by Shapiro-Wilk test. Data were analyzed using two-tailed paired *t* test (**c**, **h**, and **i**) and the Mantel-Cox test (**e** and **g**), and were presented as mean ± s.e.m.

**
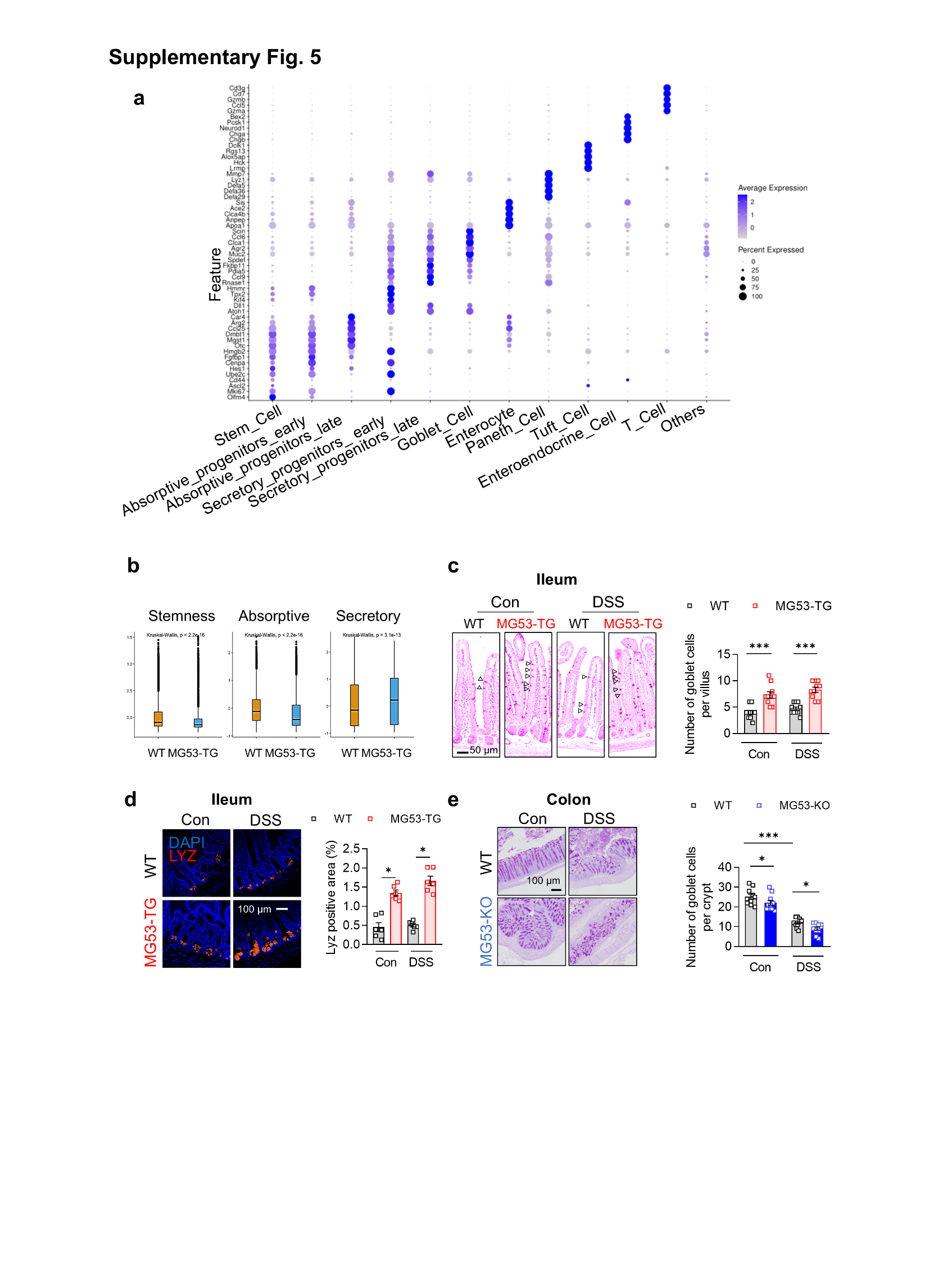
**

**Fig. S5.** MG53 promotes secretory lineage commitment of ISCs. **a** Expression of marker genes of each cell cluster. **b** The stemness, absorptive, and secretory scores of Epcam^+^CD45^-^ cells derived from MG53-TG and WT controls. **c** Representative images and statistic results of PAS staining of goblet cells in the ileum of MG53-TG mice and WT controls. *n* = 10 for each group. Scale bar, 50 μm. **d** Representative images and statistic results of immunofluorescence staining of Lysozyme (Lyz, red) in the intestine of MG53-TG mice and their WT littermates. *n* = 6 for each group. Nuclei were stained with DAPI (blue); scale bar, 100 μm. **e** Representative images and statistic results of PAS staining of goblet cells in the colon of MG53-KO mice and WT controls. *n* = 10 for each group. Scale bar, 100 μm. Data were analyzed using Kruskal-Wallis test (**b**) and two-tailed paired *t* test (**c**, **d**, and **e**), and were presented as mean ± s.e.m. **P* < 0.05 and ****P* < 0.001 as compared with the corresponding normal tissues.

**
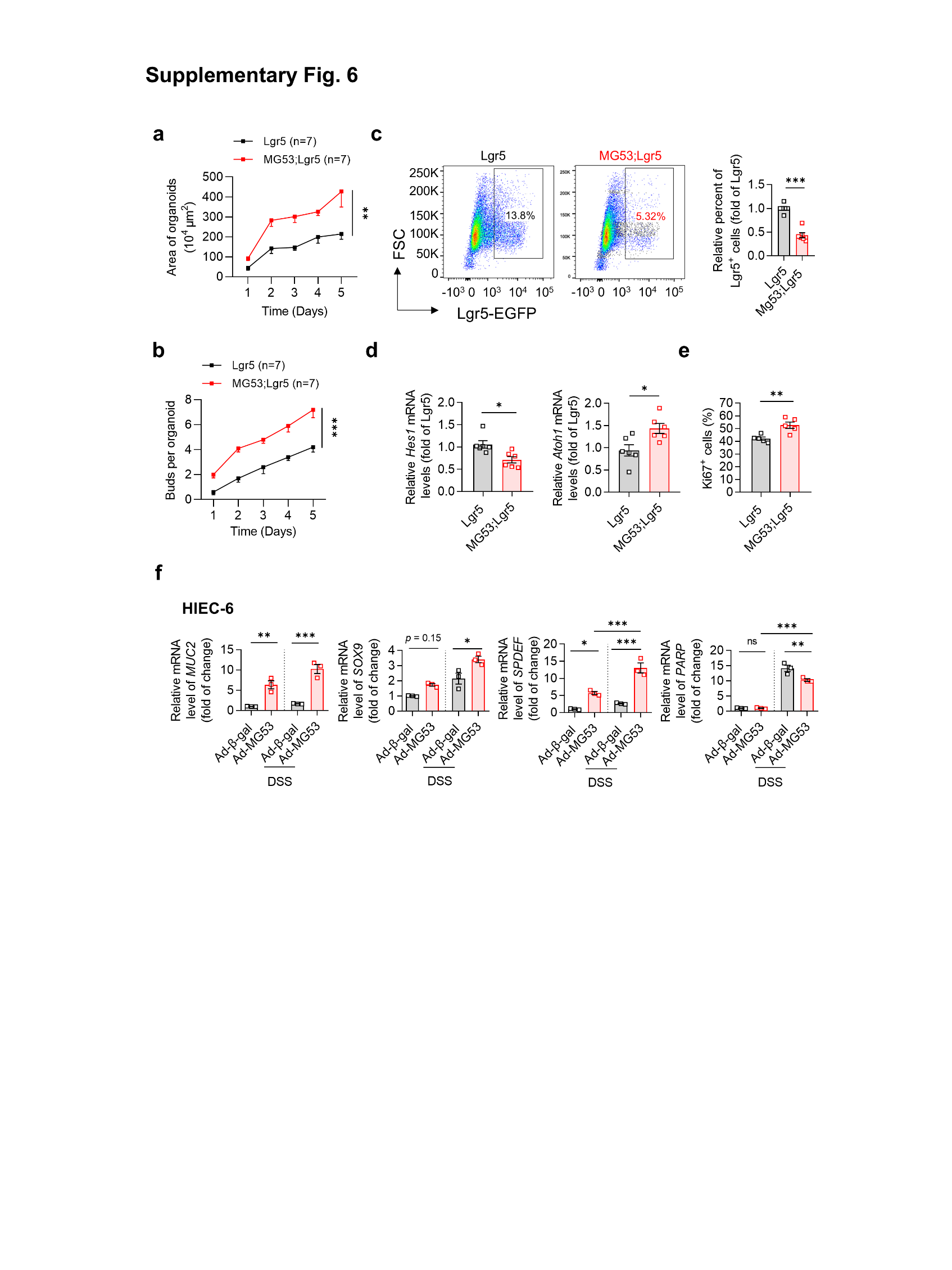
Fig. S6.** MG53 promotes the differentiation of ISCs towards secretory lineage. **a-b** Statistic results of the area of organoids (**a**) and number of buds (**b**) of the intestinal organoids derived from MG53;Lgr5 mice and their Lgr5 littermates at the indicated time points. *n* = 7 for each group. **c** Representative FACS profiles and statistic results of Lgr5^+^ cells in the MG53;Lgr5 and the control Lgr5 mice. *n* = 6 for each group. **d** Relative mRNA levels of *Hes1* and *Atoh1* in the colonic organoids derived from MG53;Lgr5 and the control Lgr5 mice. *n* = 6 for each group. **e** Statistic results of FACS profiles of Ki-67^+^ cells in colonic organoids derived from MG53;Lgr5 and the control Lgr5 mice. *n* = 5 for each group. **f** Relative mRNA levels of *MUC2*, *SOX9*, *SPDEF* and *PARP* in HIEC-6 cells after DSS treatment. *n* = 3 for each group. Ad-β-gal and Ad-MG53, cells infected with adenovirus containing expression vector of β-gal and MG53, respectively. Normal distribution was confirmed by Shapiro-Wilk test. Data were analyzed using Kruskal-Wallis test (**a** and **b**) and two-tailed paired *t*-test (**c**, **d**, **e**, and **f**), and were presented as mean ± s.e.m. ns, not significant; **P* < 0.05, ***P* < 0.01, and ****P* < 0.001 as compared with the corresponding normal tissues.

**
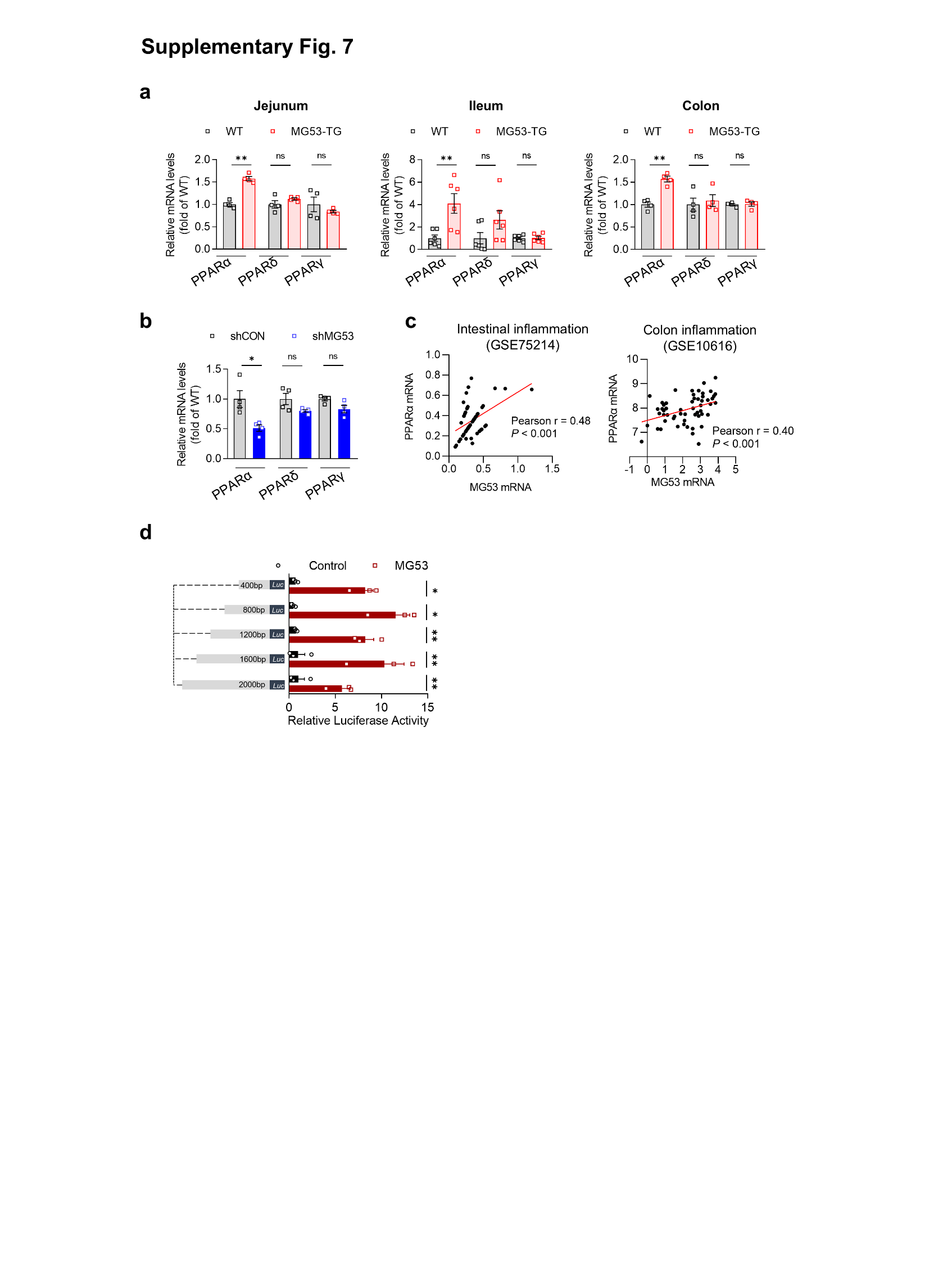
**

**Fig. S7.** MG53 upregulates the expression of PPARα. **a** Relative intestinal mRNA levels of PPAR family members in the jejunum, ileum, and colon of MG53-TG and WT controls. For the jejunum and colon, *n* = 6 for each group; for the ileum, *n* = 4 for each group. **b** Relative mRNA levels of PPAR family members in the HCT116 cells with or without inhibiting the expression of MG53 by specific shRNA targeting MG53. *n* = 4 for each group. shCON and shMG53 were HCT116 cells infected with adeno-associated virus expressing control shRNA and shRNA targeting MG53, respectively. **c** The Pearson correlation analysis of the mRNA levels of MG53 and PPARα in patients (GEO: GSE75214; *n* = 50) and (GEO: GSE10616; *n* = 58). **d** Luciferase reporter activity driven by the PPARα promoter with MG53 overexpression in the HIEC-6 cells. *n* = 3 for each group. Normal distribution was confirmed by Shapiro-Wilk test. Data were analyzed using two-tailed *t* test (**a**, **b**, and **d**) and Pearson correlation analysis (**c**), and were presented as mean ± s.e.m. ns, not significant, **P* < 0.05 and ***P* < 0.01 as compared with the corresponding controls.

**
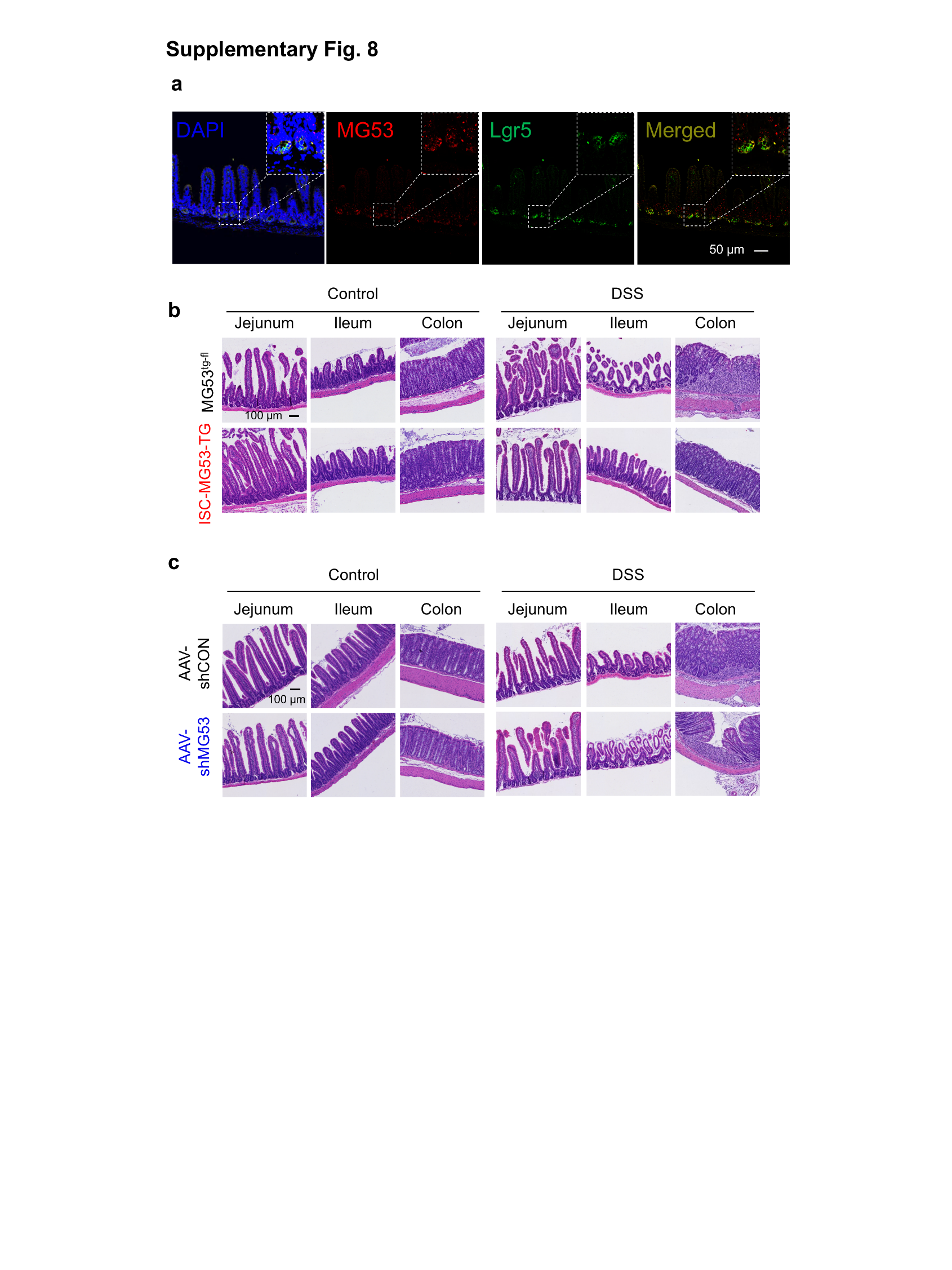
Fig. S8. a** Representative RNAscope images of MG53 (red) and Lgr5 (green) of the murine intestine. Nuclei were stained with DAPI (blue). Scale bar, 50 μm. **b** Representative H&E staining images of the jejunum, ileum, and colon of ISC-MG53-TG and MG53^tg-fl^ mice. Scale bar, 100 μm. **c** Representative H&E staining images of the jejunum, ileum, and colon of the Lgr5 mice injected with AAV-shMG53 or control AAV-shCON. Scale bar, 100 μm.

**
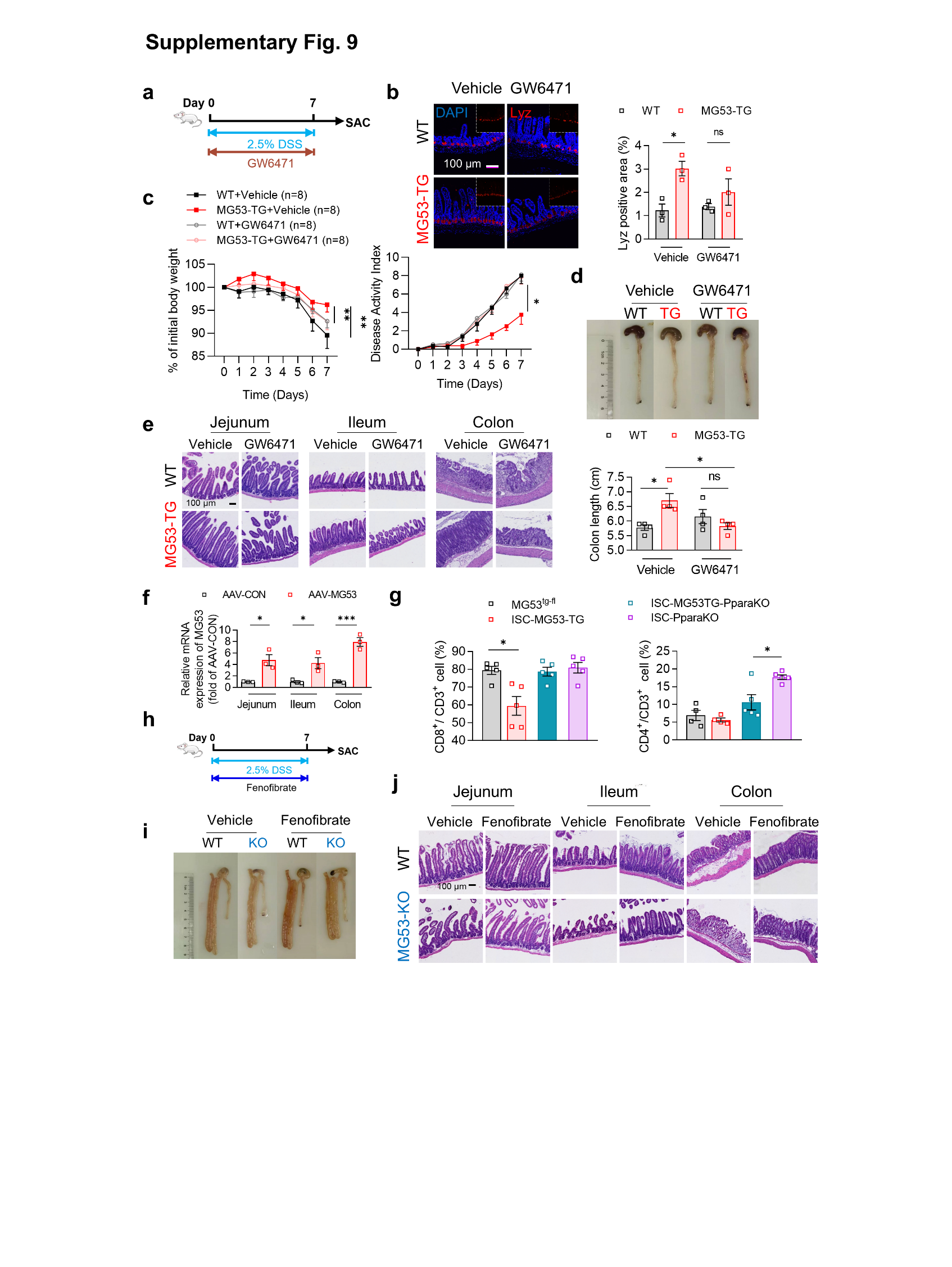
**

**Fig. S9.** PPARα is both necessary and sufficient in mediating the protective effects of MG53 in response to DSS treatment. **a** Experimental design of GW6471 treatment in mice subjected to DSS challenge. **b** Representative images and statistic results of immunofluorescence staining of Lysozyme (Lyz, red) in the intestine of MG53-TG mice and their WT littermates on day 7 after DSS treatment and treated with GW6471 or vehicle. *n* = 3 for each group. Nuclei were stained with DAPI (blue); scale bar, 100 μm. **c-e** Body weight change and DAI (**c**; n = 8 for each group), representative images and statistic results of colon length (**d**; n = 4 for each group), and representative H&E staining images of the jejunum, ileum, and colon (**e**) of MG53-TG mice and their WT littermates treated with GW6471 or vehicle following DSS challenge. Scale bar, 100 μm. **f** Relative mRNA levels of MG53 in different intestine segments after AAV-MG53 injection. *n* = 3 for each group. **g** Statistic results of FACS profiles of indicated immune cell populations in the intestine of MG53^tg-fl^, ISC-MG53-TG, ISC-Ppara-KO, and ISC-MG53TG-PparaKO mice on day 7 of DSS treatment. *n* = 5 for each group. **h** Experimental design of Fenofibrate treatment in the mice subjected to DSS challenge. **i-j** Representative images of the intestine (**i**) and H&E staining images of the jejunum, ileum, and colon (**j**; scale bar, 100 μm.) of MG53-KO (KO) and their WT littermates treated with fenofibrate or vehicle following DSS challenge. Normal distribution was confirmed by Shapiro-Wilk test. Data were analyzed using two-tailed paired *t* test (**b**, **d**, **f**, and **g**) and one-way ANOVA with Tukey post hoc test (**c**), and were presented as mean ± s.e.m. ns, not significant, **P* < 0.05, ***P* < 0.01, and ****P* < 0.001 as compared with the corresponding normal tissues.

**
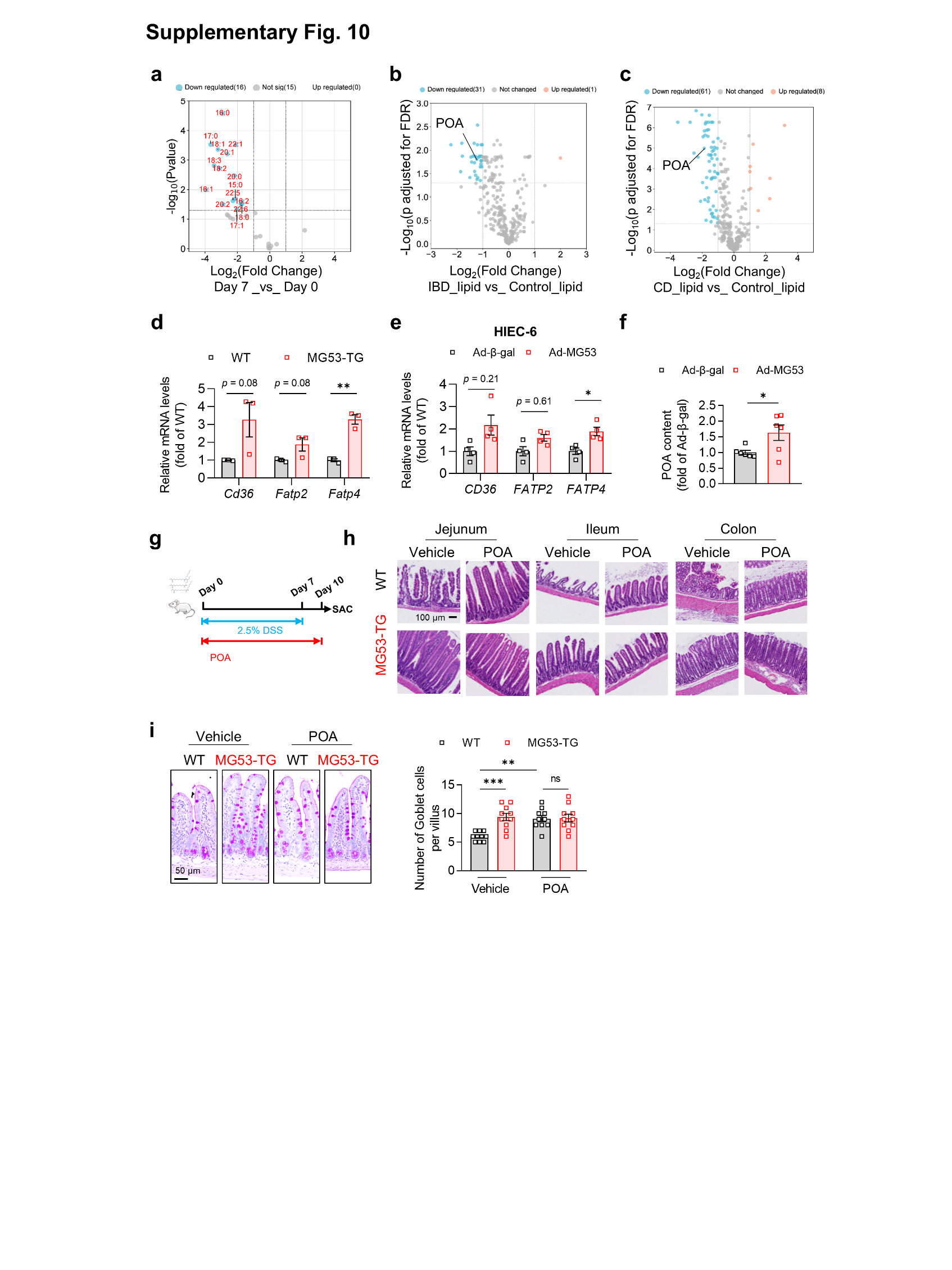
Fig. S10.** POA treatment mitigates intestinal damage induced by DSS. **a** Volcano plots of intestinal lipidomic analysis of free fatty acids (FFA) after 7-day DSS treatment (Day 7) vs. baseline (Day 0).*n* = 3 for each group. **b-c** Volcano plots of intestinal lipidomic analysis in healthy human (Control, *n* = 20) vs. patients with IBD (*n* = 40) (**b**) or vs. patients with Crohn’s disease (CD, *n* = 20) (**c**) using online dataset. POA, palmitoleic acid. **d** Relative intestinal mRNA levels of *Cd36*, *Fatp2*, and *Fatp4* in MG53-TG and WT littermates. *n* = 3 for each group. **e** Relative intestinal mRNA levels of *CD36*, *FATP2*, and *FATP4* in HIEC-6 cells with or without overexpression of MG53 by adenoviral infection. *n* = 4 for each group. **f** POA content in HIEC-6 cells after incubated with 100 μM POA for 5 min, with or without overexpression of MG53 by adenoviral infection. *n* = 6 for each group. Ad-β-gal and Ad-MG53, cells infected with adenovirus containing expression vector of β-gal and MG53, respectively. **g** Experimental design for POA treatment in mice. **h** Representative H&E staining images of the jejunum, ileum, and colon of MG53-TG and WT littermates treated with POA or vehicle following DSS challenge. Scale bar, 100 μm. **i** Representative images and statistic results of PAS staining of goblet cells in the ileum of MG53-TG and WT mice with or without POA treatment. *n* = 10 for each group. Scale bar, 50 μm. Normal distribution was confirmed by Shapiro-Wilk test. Data were analyzed using two-tailed *t* test (**d,** **e**, **f**, and **i**) and were presented as mean ± s.e.m. ns, **P* < 0.05, ***P* < 0.01, and ****P* < 0.001 as compared with the corresponding controls.

**
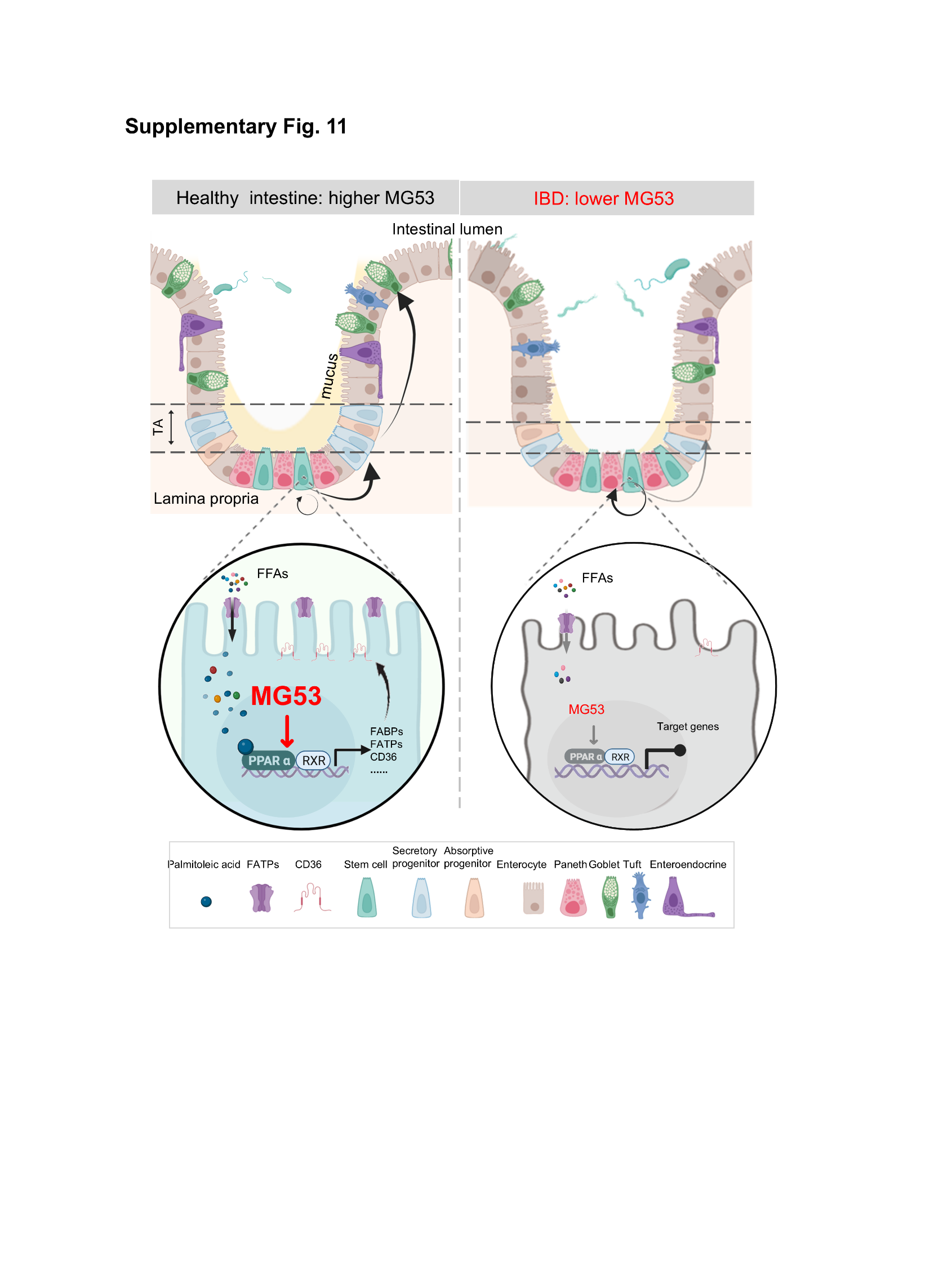
**

**Figure. S11.** Schematic diagram of MG53 alleviating intestinal injury via activation of PPARα signaling. Created with *BioRender.com*. MG53 induces the expression of PPARα in the ISCs. PPARα subsequently upregulates the expression of fatty acids transporters and increased fatty acid content. These fatty acids serve as PPARα agonists to further enhance PPARα activity. The enhanced PPARα signaling promotes biased differentiation of ISCs towards secretory lineage and facilitate intestinal damage repair.

**Table S1 Patient information**

| **Number** | **Sample type** | **Gender** | **Age** | **Stage** |
| --- | --- | --- | --- | --- |
| 1 | Serum | M | 63 | remission |
| 2 | Serum | M | 63 | remission |
| 3 | Serum | F | 60 | remission |
| 4 | Serum | F | 43 | remission |
| 5 | Serum | F | 35 | remission |
| 6 | Serum | M | 60 | remission |
| 7 | Serum | F | 33 | remission |
| 8 | Serum | M | 36 | remission |
| 9 | Serum | F | 45 | remission |
| 10 | Serum | F | 48 | remission |
| 11 | Serum | F | 31 | remission |
| 12 | Serum | F | 37 | IBD |
| 13 | Serum | M | 23 | IBD |
| 14 | Serum | M | 45 | IBD |
| 15 | Serum | F | 55 | IBD |
| 16 | Serum | F | 36 | IBD |
| 17 | Serum | F | 33 | IBD |
| 18 | Serum | M | 60 | IBD |
| 19 | Serum | M | 42 | IBD |
| 20 | Serum | M | 32 | IBD |
| 21 | Serum | F | 59 | IBD |
| 22 | Serum | M | 67 | IBD |
| 23 | Serum | M | 32 | IBD |
| 24 | Serum | M | 27 | IBD |
| 25 | Serum | F | 22 | IBD |
| 26 | Serum | F | 30 | IBD |
| 27 | Serum | M | 33 | IBD |
| 28 | Serum | M | 24 | IBD |
| 29 | Serum | M | 32 | IBD |
| 30 | Serum | F | 51 | IBD |
| 31 | Serum | M | 44 | IBD |
| 32 | Serum | M | 68 | IBD |
| 33 | Serum | M | 65 | IBD |
| 34 | Serum | F | 34 | IBD |
| 35 | Serum | M | 28 | IBD |
| 36 | Serum | M | 18 | IBD |
| 37 | Serum | F | 50 | IBD |
