# Supplementary material for: Mitsugumin 53 drives stem cell differentiation easing intestinal injury and inflammation
Source: Signal Transduct Target Ther. 2025 Jun 11;10:183. doi: 10.1038/s41392-025-02268-x (PMC12152187; doi:10.1038/s41392-025-02268-x)

Supplementary Fig. 1a

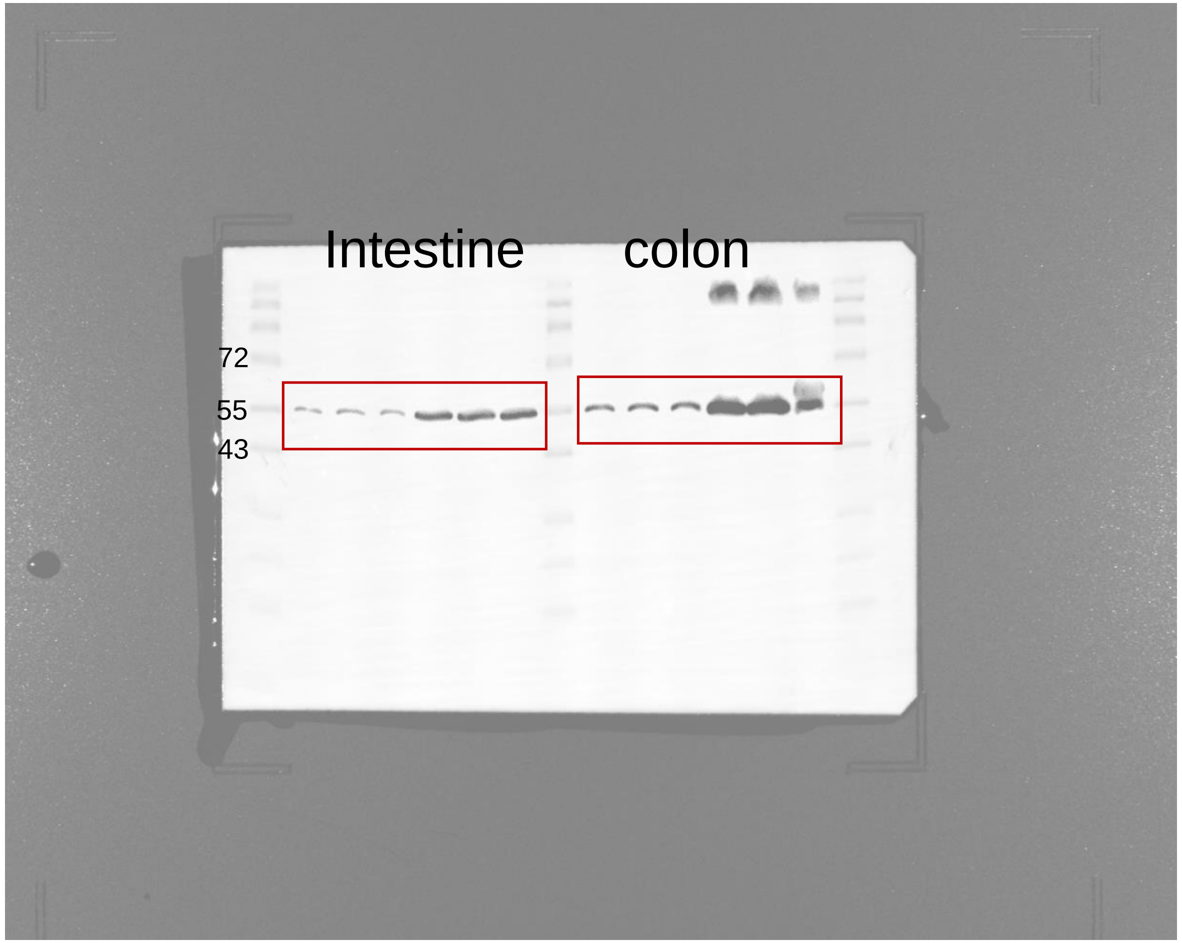

Supplementary Fig. 1a

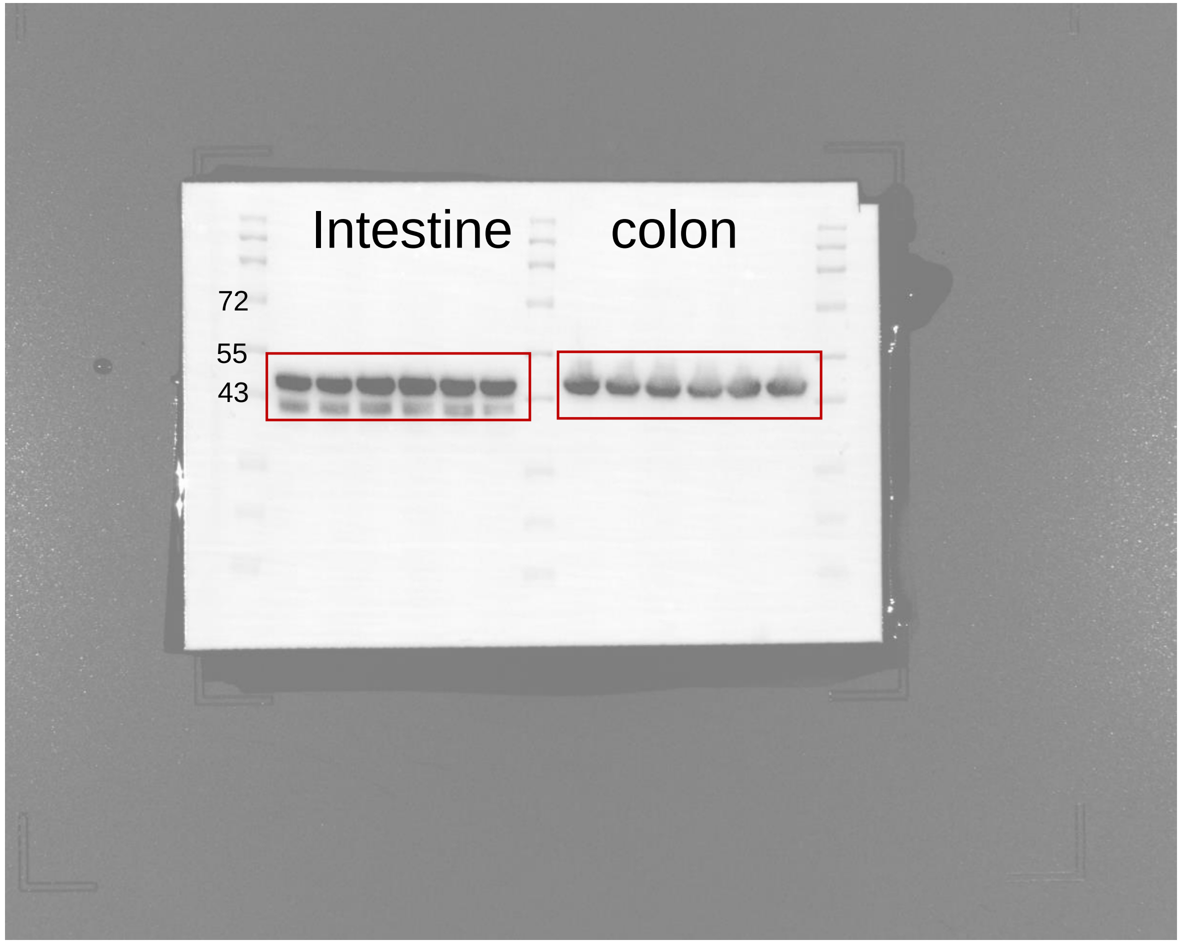

Supplementary Fig. 2a

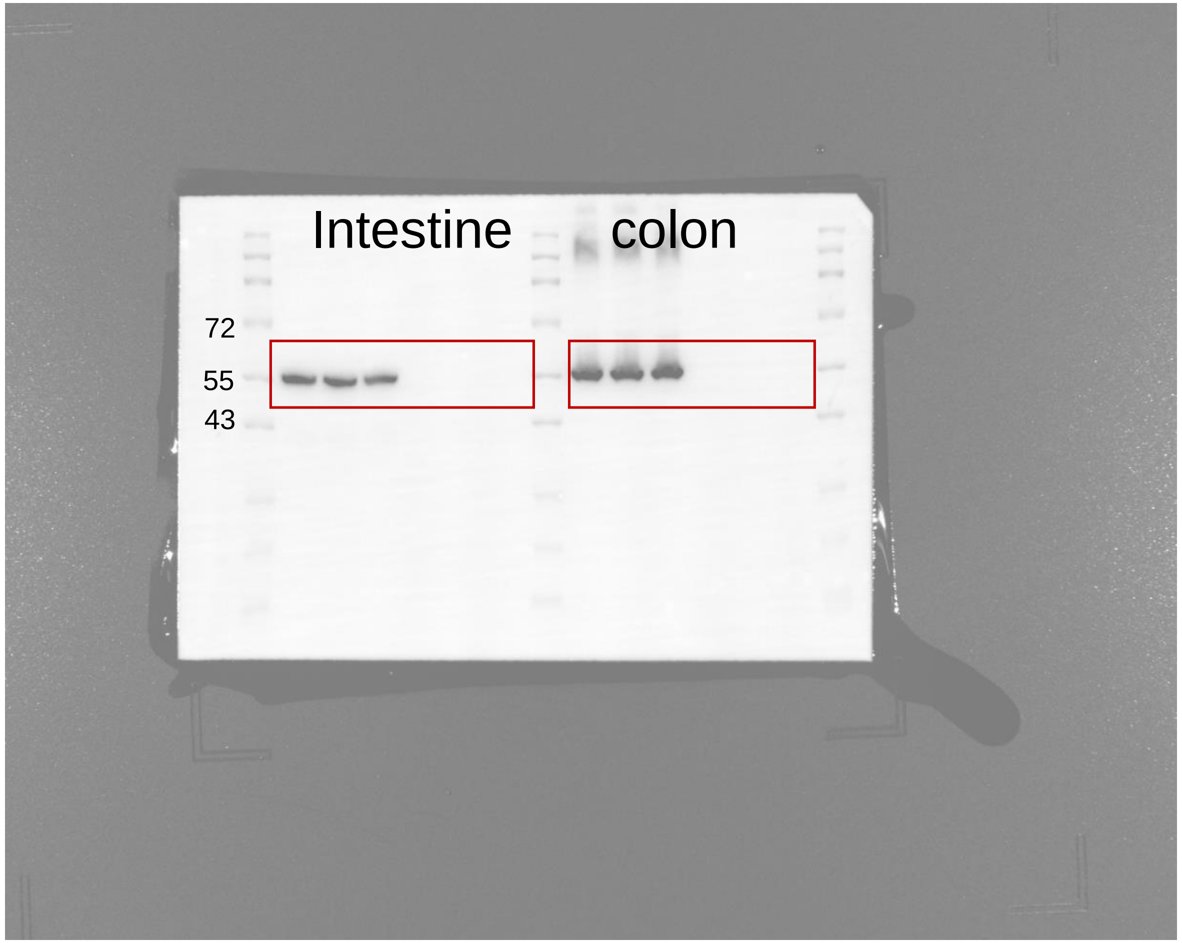

Supplementary Fig. 2a

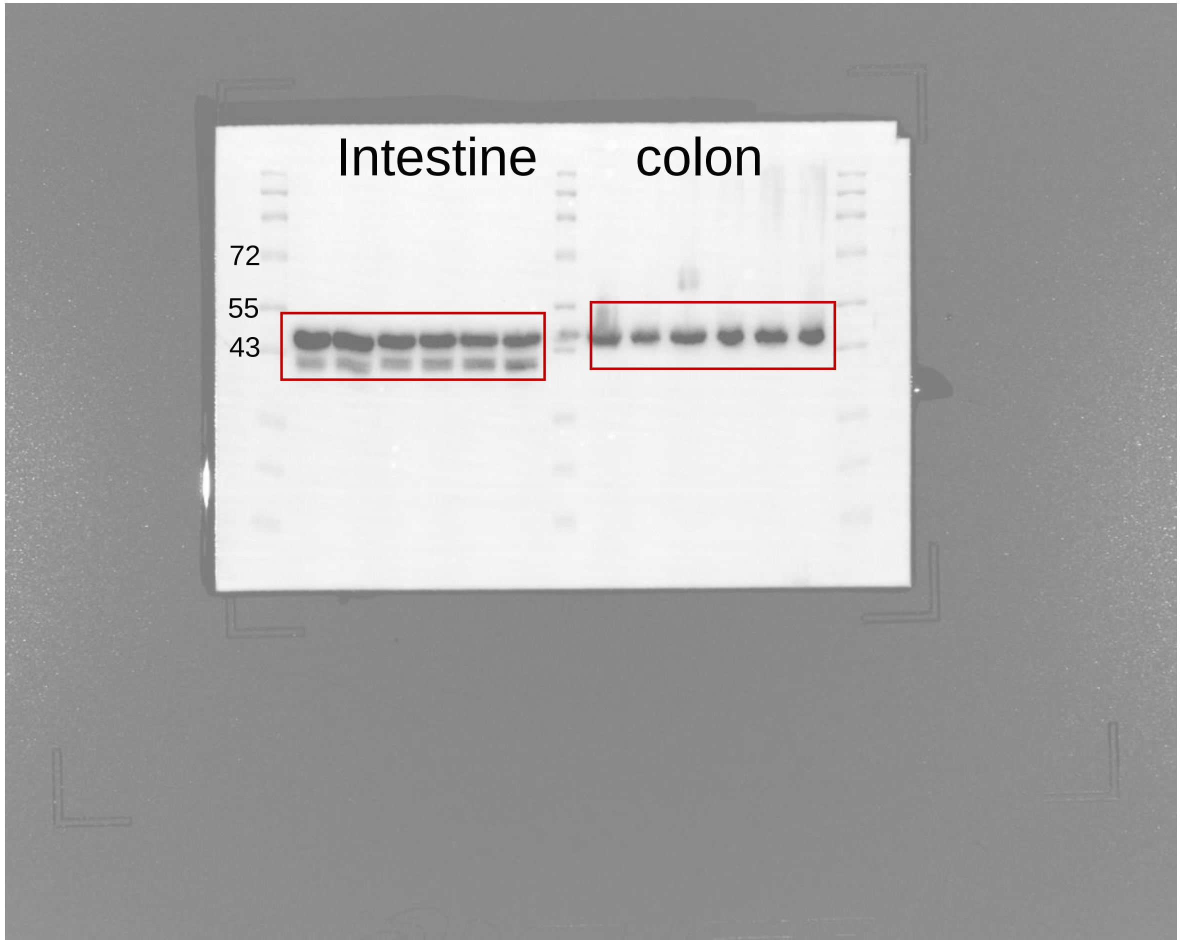

Figure 2h

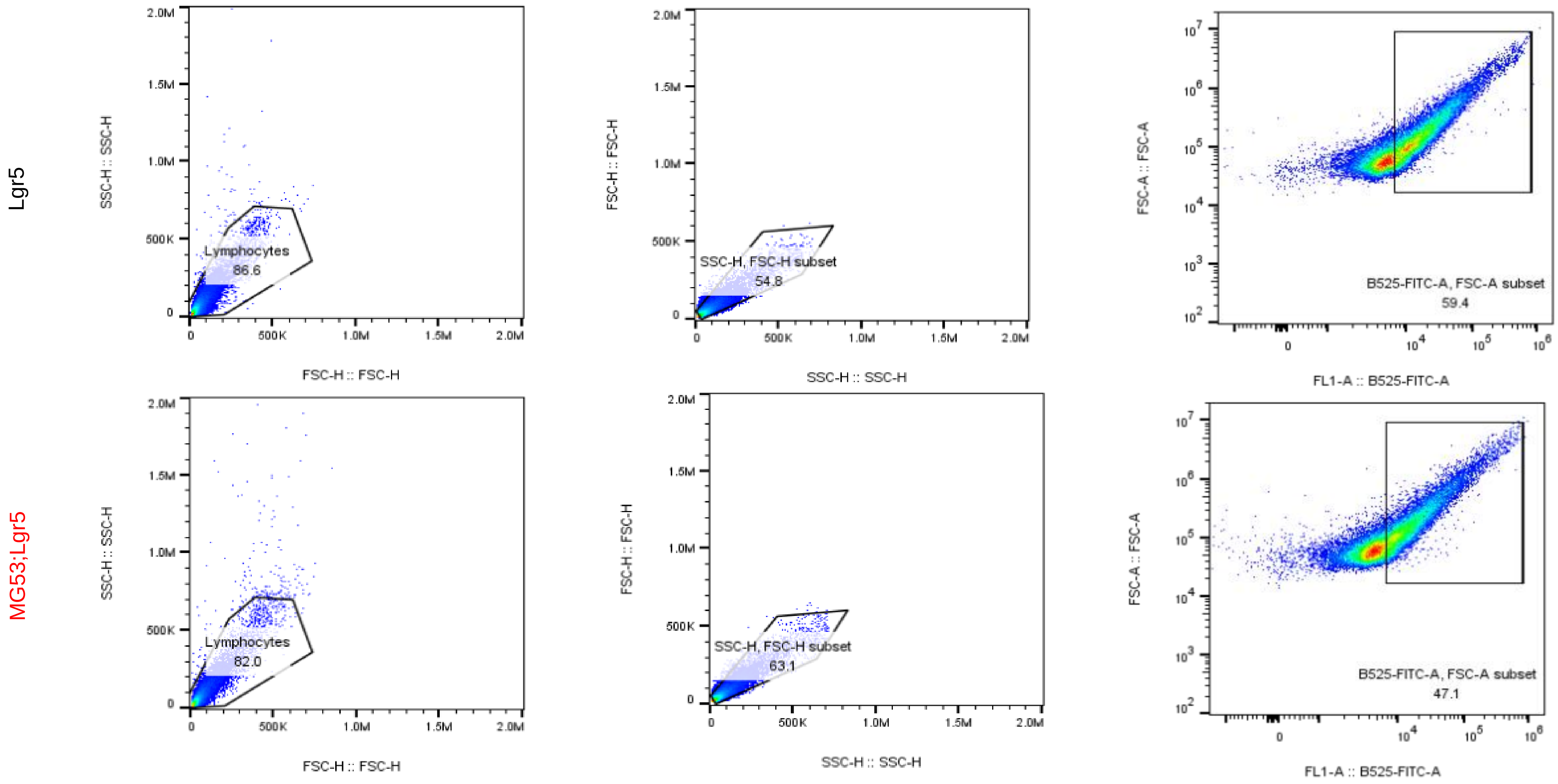

Lgr5
MG53;Lgr5
SSC-H :: SSC-H
FSC-H :: FSC-H
Lymphocytes
86.6
82.0
SSC-H, FSC-H subset
54.8
63.1
FSC-A :: FSC-A
FL1-A :: B525-FITC-A
B525-FITC-A, FSC-A subset
59.4
47.1

Supplementary Figure 6c

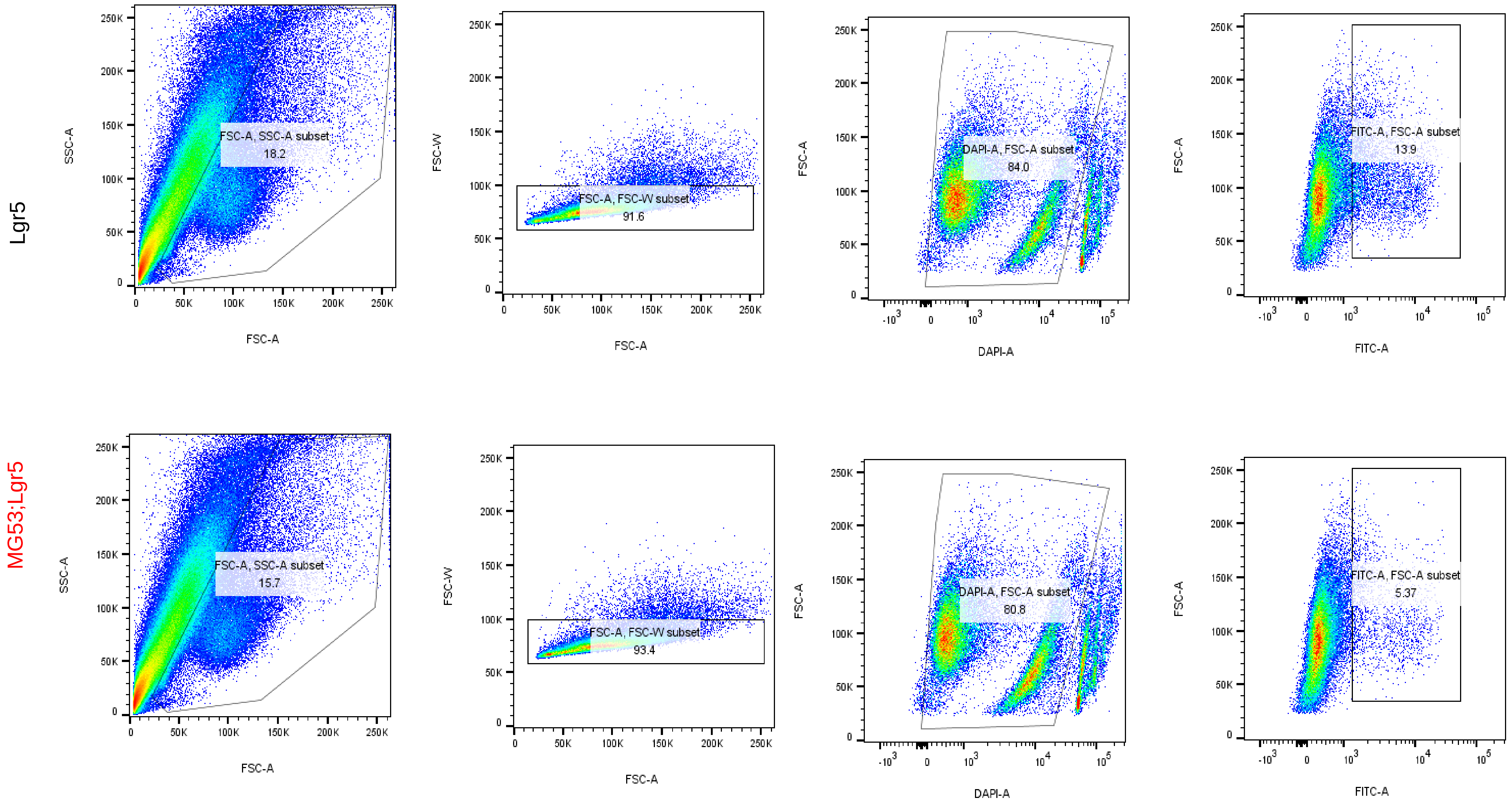

Supplementary Figure 6e

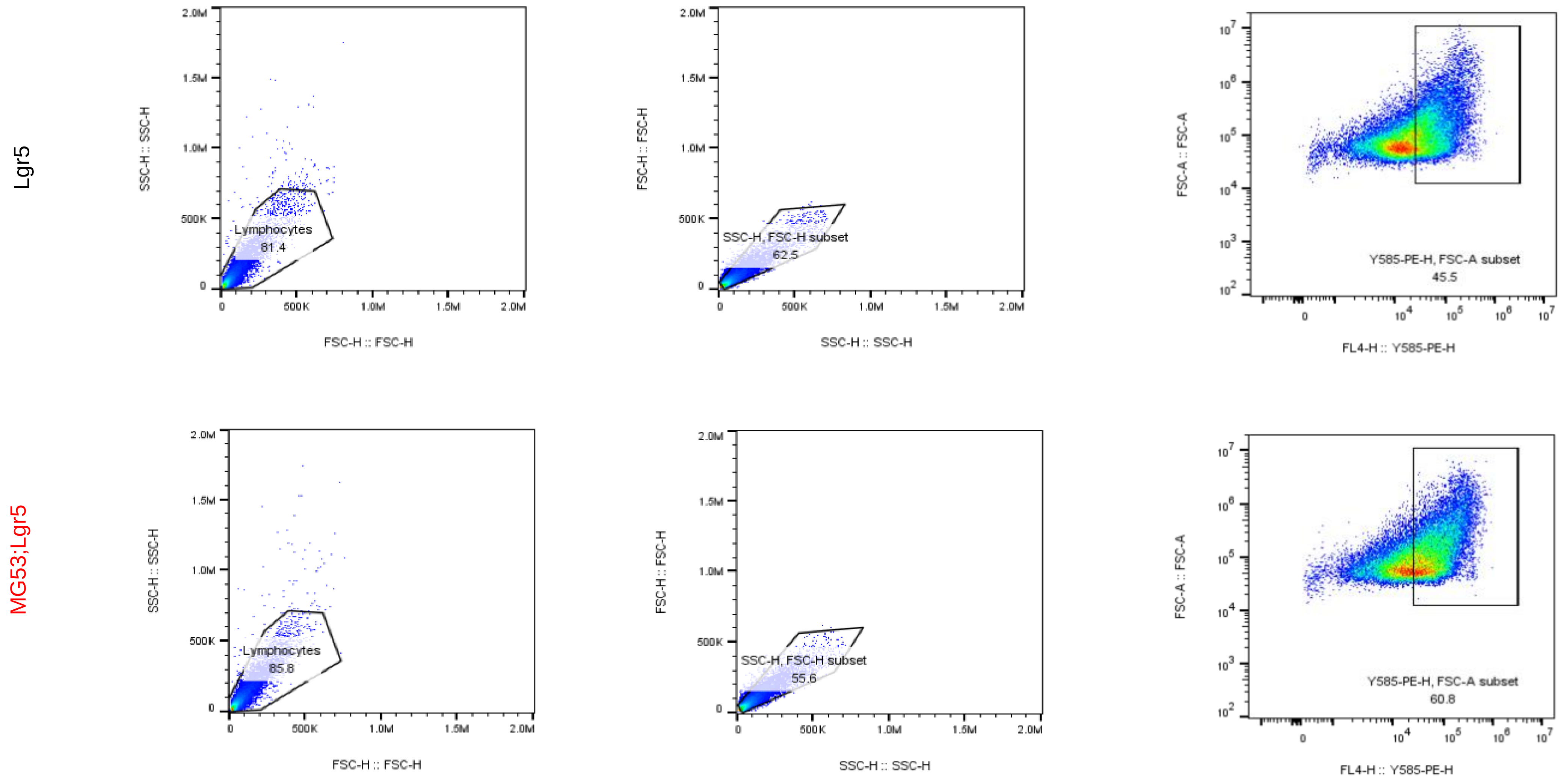

Supplementary Figure 9g

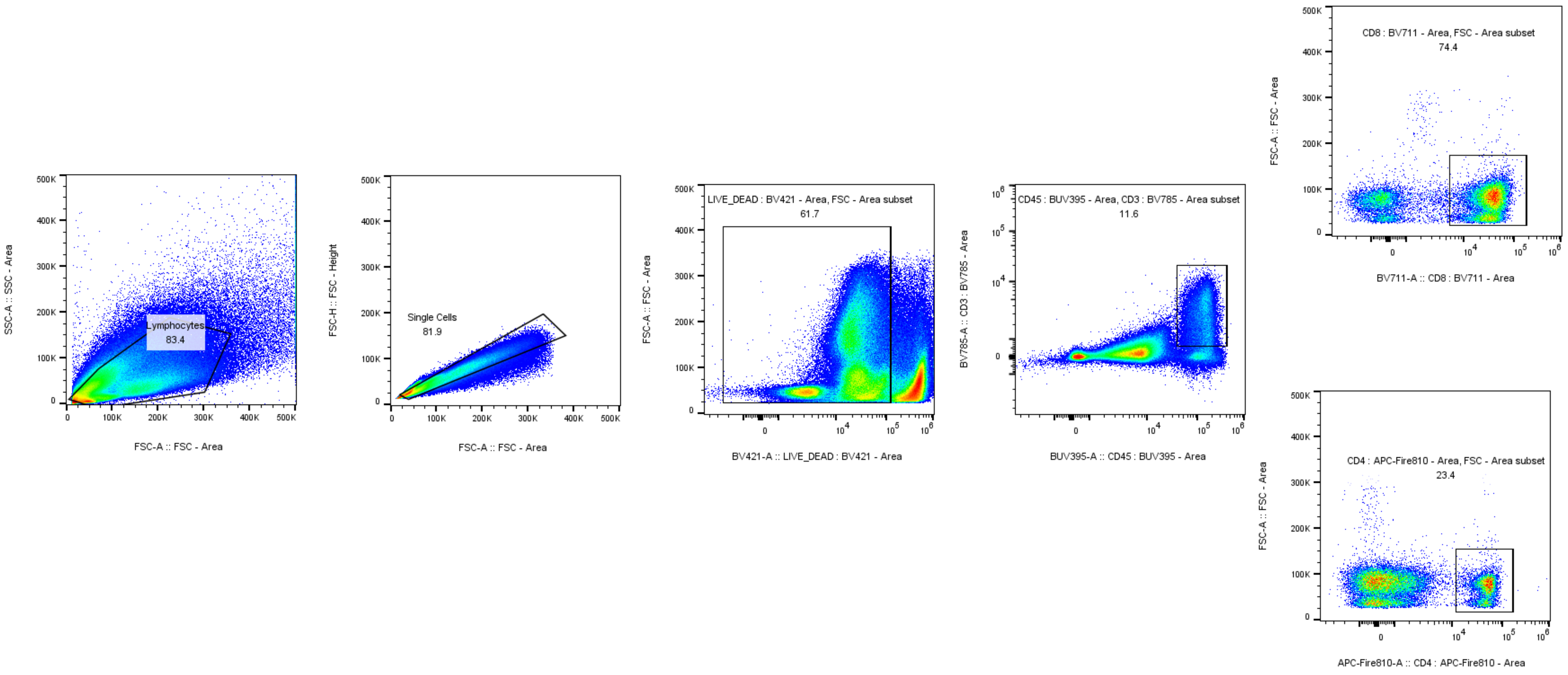

Supplement: Supplementary file 2 — 20250430_MG53 in IBD_Raw WB and FACS gating info [file 41392_2025_2268_MOESM2_ESM.pdf]
